# Crystallographic models of SARS-CoV-2 3CL^pro^: in-depth assessment of structure quality and validation

**DOI:** 10.1107/S2052252521001159

**Published:** 2021-02-09

**Authors:** Mariusz Jaskolski, Zbigniew Dauter, Ivan G. Shabalin, Miroslaw Gilski, Dariusz Brzezinski, Marcin Kowiel, Bernhard Rupp, Alexander Wlodawer

**Affiliations:** a Department of Crystallography, Faculty of Chemistry, A. Mickiewicz University, Poznan, Poland; b Center for Biocrystallographic Research, Institute of Bioorganic Chemistry, Polish Academy of Sciences, Poznan, Poland; c Center for Structural Biology, National Cancer Institute, Frederick, MD 21702, USA; d Department of Molecular Physiology and Biological Physics, University of Virginia, Charlottesville, VA 22908, USA; e Institute of Computing Science, Poznan University of Technology, Poznan, Poland; f k.-k Hofkristallamt, San Diego, CA 92084, USA; g Institute of Genetic Epidemiology, Medical University Innsbruck, A-6020 Innsbruck, Austria

**Keywords:** COVID-19, SARS-CoV-2, coronavirus, structure-guided drug discovery, ligand validation, viral proteases, Protein Data Bank, reproducibility

## Abstract

The appearance at the end of 2019 of the new SARS-CoV-2 coronavirus led to an unprecedented response by the structural biology community, resulting in the rapid determination of many hundreds of structures of proteins encoded by the virus. As part of an effort to analyze and, if necessary, remediate these structures as deposited in the Protein Data Bank (PDB), this work presents a detailed analysis of 81 crystal structures of the main protease 3CL^pro^, an important target for the design of drugs against COVID-19. The structures of the unliganded enzyme and its complexes with a number of inhibitors were determined by multiple research groups using different experimental approaches and conditions; the resulting structures span 13 different polymorphs representing seven space groups. The structures of the enzyme itself, all determined by molecular replacement, are highly similar, with the exception of one polymorph with a different inter-domain orientation. However, a number of complexes with bound inhibitors were found to pose significant problems. Some of these could be traced to faulty definitions of geometrical restraints for ligands and to the general problem of a lack of such information in the PDB depositions. Several problems with ligand definition in the PDB itself were also noted. In several cases extensive corrections to the models were necessary to adhere to the evidence of the electron-density maps. Taken together, this analysis of a large number of structures of a single, medically important protein, all determined within less than a year using modern experimental tools, should be useful in future studies of other systems of high interest to the biomedical community.

## Abstract 2

Over 100 models of SARS-CoV-2 3CL^pro^, a major drug-design target for COVID-19, have been carefully validated and assembled in a dedicated database. Their comparative analysis provides lessons for similar medically oriented efforts and for structural biology in general.

## 1. Introduction   

The appearance at the end of 2019 of the new SARS-CoV-2 coronavirus, the causative agent of COVID-19, led to an unprecedented response by the scientific community. Structural biologists were among the first to swing into action, with the first structure of a SARS-CoV-2 protein being deposited in the Protein Data Bank (PDB; Berman *et al.*, 2000 ▸) within two weeks of the release of the genomic sequence of the virus. This was the structure of the main cysteine protease (PDB entry 6lu7; Jin, Zhao *et al.*, 2020 ▸), varyingly named 3CL^pro^ or M^pro^ and sometimes NSP5. This first model was followed by 80 PDB depositions (as of 15 October 2020) of crystal structures of the same enzyme in the free apo form (Table 1 ▸) and in complex with a variety of both covalently and noncovalently bound inhibitors (Table 2 ▸). (See also the computer-searchable Supplementary Table S1.) The structures were obtained using different X-ray sources (synchrotrons, rotating-anode generators in home laboratories and X-ray free-electron lasers), as well as with neutrons from a spallation source. Some structures of identical complexes were determined in different space groups, sometimes under different conditions, and/or by different research groups. In addition, structures of 115 potential ligand complexes of 3CL^pro^ determined in large-scale ligand fragment-screening campaigns assisted by the *Pan-Dataset Density Analysis* (*PanDDA*; Pearce, Krojer, Bradley *et al.*, 2017 ▸) methodology have been deposited. In the following, 3CL^pro^ refers to the enzyme from SARS-CoV-2, while homologous enzymes from other coronaviruses are identified fully with the names of the parent viruses.

The availability of a large number of structures of a single protein, all determined within less than a year from data collected using modern radiation sources and refined with similar modern software, provides a unique opportunity to investigate the limits of accuracy versus inherent structural variability of a single protein target. Some attempts to collect and analyze a large assembly of structures of a single protein have been reported in the past (for example, for the protease encoded by the human immunodeficiency virus; Vondrasek *et al.*, 1997 ▸; Vondrasek & Wlodawer, 2002 ▸), but no detailed assessment of their relative quality and true differences was performed. There have been specific campaigns to validate and correct PDB models of medicinally important drug-design targets, for instance metallo-β-lactamases (Raczynska *et al.*, 2018 ▸) or proteins that bind cisplatin (Shabalin *et al.*, 2015 ▸). The analysis, verification and improvement (if necessary) of all crystal structures of SARS-CoV-2 proteins is already the subject of an ongoing project (Wlodawer *et al.*, 2020 ▸; Brzezinski *et al.*, 2021 ▸), but without a dedicated overview and comparison of different structures. It is our aim to present here such a detailed analysis for 3CL^pro^ that may be useful not only for this particular protein target but also to guide any future projects aimed at the interpretation of multiple structures of medicinally important macromolecules.

## 2. The role and significance of 3CL^pro^   

As is typical for coronaviruses, the genome of SARS-CoV-2 encodes two large viral polyproteins, pp1a and pp1ab, which need to be processed before an active virion can be reconstituted. This task is accomplished by two virally encoded cysteine proteases: the chymotrypsin-like main protease (3CL^pro^) and papain-like protease (PL^pro^). The absolute requirement for the activity of 3CL^pro^ for viral replication has been demonstrated for other coronaviruses through mutagenesis experiments (Kim *et al.*, 1995 ▸; Stobart *et al.*, 2012 ▸). 3CL^pro^ proteolytically processes the viral polyproteins at 11 junctions, generating the individual proteins critical for virus replication, including autoprocessing itself (Hegyi & Ziebuhr, 2002 ▸). It is important to note that there are no close human analogs of coronaviral 3CL^pro^, and thus interference with the activity of this enzyme is not likely to lead to serious side effects, as was previously postulated for the very closely related protease from SARS-CoV (Anand *et al.*, 2003 ▸). Inhibition of the activity of viral proteases has been shown to be of practical therapeutic importance for diseases such as those caused by human immunodeficiency virus (HIV; Wlodawer & Vondrasek, 1998 ▸) and hepatitis C virus (HCV; Bacon *et al.*, 2011 ▸). Since 3CL^pro^ is a cysteine protease, albeit with a fold related to the serine protease chymotrypsin, there is a good chance that specific inhibitors of this enzyme could be developed, making 3CL^pro^ an important drug target for antivirals. These concepts have been pursued in the past for SARS-CoV 3CL^pro^ (Pillaiyar *et al.*, 2016 ▸), although the rapid disappearance of that virus decreased interest in such work, and they have now been proposed again (Konwar & Sarma, 2021 ▸).

## 3. Three-dimensional structure and the active site of 3CL^pro^   

### 3.1. 3CL^pro^ dimer   

**Figure 1:**
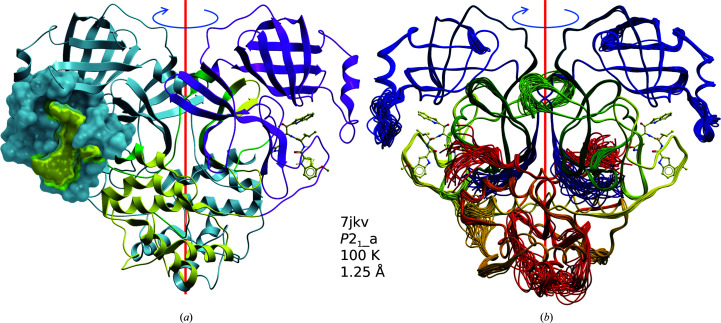
High-resolution structure and dynamics of SARS-CoV-2 3CL^pro^, exemplified by PDB entry 7jkv (1.25 Å resolution) from the *P*2_1__a polymorph (Table 2 ▸). (*a*) Cartoon model of the dimer with the vertical twofold NCS axis (red line) in the plane of the paper. In the left protomer (light blue), the binding-pocket surface (blue surface) around the covalently bound inhibitor GRL2420 (yellow surface) is highlighted. In the right protomer, the catalytic domain is highlighted in purple, residues involved in dimer contacts are colored green and the remaining regions are in yellow. (*b*) Visualization of protein plasticity through an ensemble of 25 molecular-dynamics traces obtained from multi-conformer refinement with *Phenix* (Burnley *et al.*, 2012 ▸). The backbone ‘worms’ of the models are colored from the N-terminus (blue) to the C-terminus (red). The relative rigidity of the binding pocket is clearly visible compared with regions of increased anisotropic movement such as some loops in the catalytic domain, parts of the C-terminal regions (orange to red) and the N- and C-­termini. N-terminal tags are common and are distant from crystal contacts. In contrast, in the single structure with a C-terminal His_6_ tag (PDB entry 6wtt, space group *P*3_2_21), only the C-terminus is exposed and disordered, while the N-terminal Ser1 participates in dimer contacts and in close crystal contacts, leaving no room for an N-terminal tag. This figure was generated with *ICMPro* (Abagyan *et al.*, 1994 ▸).

SARS-CoV-2 3CL^pro^ is a homodimeric protease consisting of two 306-residue polypeptide chains. Each chain folds into two domains, with the double β-barrel fold of the catalytic N-terminal domain (residues 9–197) resembling that of chymotrypsin. The dimer interface, with a buried surface area of ∼1400 Å^2^ (calculated by *PISA*; Krissinel & Henrick, 2007 ▸), is typical for an obligate dimer in solution. However, in >70% of the deposited structures the dimer twofold-symmetry axis coincides with a crystallographic dyad, and the deposited models contain only one subunit of the dimer (Table 3 ▸). The first ∼15 N-terminal residues, residues in the region 115–141 of the catalytic domain and multiple residues in the C-terminal region participate in the formation of the dimer interface [Fig. 1 ▸(*a*)]. Isolated monomers exhibit diminished levels of catalytic activity or are inactive, and dimerization inhibitors have been designed for SARS-CoV 3CL^pro^ (Barrila *et al.*, 2006 ▸) and SARS-CoV-2 3CL^pro^ (Goyal & Goyal, 2020 ▸). Although the N-terminus is involved in dimerization contacts, it is flexible and extends into the solvent, consistent with the lack of a detrimental effect of even substantial N-terminally attached expression tags, as exemplified by PDB entry 7cbt. A visualization of the structural dynamics based on translation–libration–screw (TLS) and molecular-dynamics (MD) analyses (Burnley *et al.*, 2012 ▸) is provided in Fig. 1 ▸(*b*).

### 3.2. Substrate-binding site   

Whereas the catalytic machinery of chymotrypsin comprises the canonical triad Ser195–His57–Asp102, only a dyad of residues, Cys145–His41, is responsible for the enzymatic activity of 3CL^pro^, with the possible involvement of a water molecule that plays the role of the third catalytic residue (see below). The SARS-CoV-2 enzyme shares 96% sequence identity with the previously extensively studied SARS-CoV 3CL^pro^ protein (Anand *et al.*, 2003 ▸), with 100% identity around the active site.

The substrate-binding site is distal to the dimer interface [Fig. 1 ▸(*a*)] and is readily accessible from solution. Notably, the different packing modes of the polymorphs do affect the accessibility of the binding site *in cristallo*, potentially hampering ligand soaking; however, most of the liganded crystal structures were presumably obtained from protein stock pre-incubated with the ligands. We note here that since many structures have not been described in a publication, many of the experimental details are often missing. In addition, in PDB entry 7khp crystal packing induced a reverse reaction leading to the covalent attachment of the C-terminus of one protein molecule to the catalytic site of an adjacent symmetry-related molecule (Lee *et al.*, 2020 ▸). The imposition of strict crystallographic symmetry in the most prevalent *C*2 polymorphs and others (Table 3 ▸) precludes differences between the two molecules of the dimer. Analysis of any conformational differences, however, is necessary to explain possible cooperativity or allosteric effects (Barrila *et al.*, 2006 ▸).

### 3.3. The biological function of 3CL^pro^   

**Figure 2:**
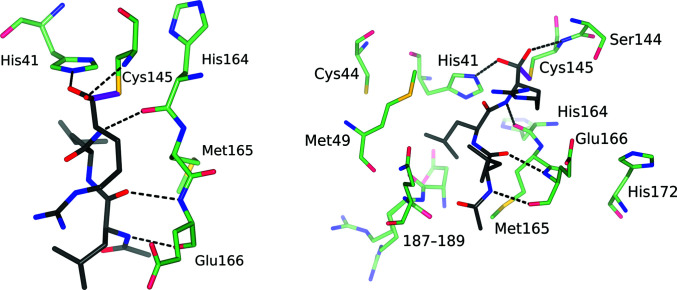
Two views of the substrate-binding pocket of 3CL^pro^. The area of interaction between a putative substrate and the enzyme is exemplified by the binding of leupeptin (PDB entry 6yz6). The leupeptin molecule in the active site is shown as sticks, with C atoms in gray, O atoms in red and N atoms in blue. 3CL^pro^ residues forming hydrogen bonds to the ligand are shown as sticks with C atoms in light green. Hydrogen bonds are shown as dashed lines. The covalent bond between leupeptin and Cys145 is shown in magenta.

The biological role of 3CL^pro^ is to first excise itself from a virally encoded polyprotein and then to cleave ten additional sequences, releasing the mature, functional viral proteins. The enzyme is specific for substrates with glutamine in the P1 position, leucine or methionine at P2 and serine, alanine or asparagine at P1′. In this nomenclature (Schechter & Berger, 1967 ▸) the peptidic ligand is presented to the active site with the sequence …P2–P1–↓–P1′–P2′…, where ↓ indicates the cleavage site and residues P*i* are docked into binding sites S*i*. As indicated by the structures of the enzyme complexed with inhibitors that retain a peptide-like character (exemplified by the tripeptide aldehyde leupeptin), the most extensive binding involves the substrate residues preceding the cleavage site. The S1 subsite of the enzyme is formed by several hydrophilic residues, including Ser144, His163, Glu166 and His172, thus resulting in a strong preference for the binding of substrates with hydrophilic P1 residues. Residue P2 docks into a largely hydrophobic cavity surrounded by Met49, Cys44 and mostly nonpolar parts of the main chain of residues 187–189. The main chain of the P3 residue forms hydrogen bonds to the main chain of Glu166 of the enzyme, corresponding to an antiparallel β-sheet motif (Fig. 2 ▸).

The water molecule that is assumed to be the equivalent of the third catalytic residue in serine proteases (by affecting the protonation state of His41 in the second step of catalysis) is nestled between the side chains of His41, His164 and Asp187 and the main-chain amide N atom of His41. This water molecule is present in all structures of the free enzyme, although sometimes it was missed and was not included in the model, leading to erroneous interpretations of its absence as being due to experimental conditions rather than incomplete modeling (Kneller, Phillips, O’Neill, Jedrzejczak *et al.*, 2020 ▸). However, this important water molecule is displaced by some inhibitors, as discussed later.

## 4. Data mining and assembly of the reference database   

The number of crystal structures of SARS-CoV-2 3CL^pro^ released by the PDB between 5 February 2020 [when the first structure, describing a complex with the inhibitor N3, became available (Jin, Du *et al.*, 2020 ▸)] and 15 October 2020, which was the cutoff date for the analysis presented here, is 196. However, most of these structures are *PanDDA* group depositions, which are not considered further here because they are not directly comparable to conventionally determined protein–ligand complex structures. *PanDDA* is, in effect, a multi-data-set map contrast-enhancement procedure that allows the placement of a known specific fragment-screening ligand into weak binding-site density (Pearce, Krojer & von Delft, 2017 ▸). The low minimum real-space correlation coefficients (RSCC > 0.7) that are considered to be useful for potential leads in *PanDDA* fragment screening are not acceptable by conventional standards (Cereto-Massagué *et al.*, 2013 ▸; Pozharski *et al.*, 2013 ▸; Wlodawer *et al.*, 2018 ▸). The mean RSCC for a set of 120 3CL^pro^-binding ligand fragments extracted from the PDB validation reports is 0.74, with an average ligand occupancy of 0.62, while for the ligand structures in Table 2 ▸ the mean RSCC is 0.88 with a mean occupancy of 0.95. Re-refinement of the provided model against the deposited data usually leads to inferior maps for weak ligands compared with the deposited maps obtained using the full *PanDDA* procedure. Routine recalculation of the event maps from the PDB-deposited *PanDDA* data is currently not feasible, and the crucial single data set for the ligand-containing structure is not (or is not consistently) provided.

Our analysis therefore concentrated on 81 individual structures that included 24 depositions of the enzyme without any ligands near the active site (Table 1 ▸) and 57 complexes with a number of different ligands, the vast majority of them being inhibitors covalently linked to the active site (Table 2 ▸). These structures were determined in 13 different polymorphs (unit cells) of seven different space groups (Table 3 ▸) from crystals grown using a variety of crystallization conditions. Experimental diffraction data were collected using several types of radiation sources (Supplementary Table S1). Since most otherwise isomorphous structures were refined by their authors with molecules located in inconsistent parts of the unit cell (or sometimes completely outside of it), all models considered here were first given a standardized placement in the unit cell with the help of the *ACHESYM* server (Kowiel *et al.*, 2014 ▸). The final structures (some of which had to be further refined) were deposited in the database at https://covid-19.bioreproducibility.org (Brzezinski *et al.*, 2021 ▸).

Almost half of all structures (36) correspond to crystals in space group *C*2 with approximate unit-cell parameters *a* = 114, *b* = 53, *c* = 45 Å, β ≃ 101° (polymorph *C*2_a) and with one protein molecule in the asymmetric unit. In these crystal structures two protein chains form the functional dimer with *C*_2_ symmetry through the operation of the crystallographic twofold axis. Six of these isomorphous structures were described by two different research groups in the equivalent, but nonstandard, space group *I*2, introducing confusion and making comparison more difficult. For the purpose of the work described here, the diffraction data for the *I*2 structures (PDB entries 6zru, 6xch, 6xqu, 6xb0, 6xb1 and 6xb2) were reindexed to the standard *C*2 setting by the operation (−*h* − *l*, *k*, *h*), with appropriate transformation of the atomic coordinates. Thus, this whole series of structures, some of which were duplicative, could be superposed and analyzed together with their electron density directly, making their comparison much easier.

The next largest group of structures (16) are also in space group *C*2, but with approximate unit-cell parameters *a* = 98, *b* = 81, *c* = 52 Å, β ≃ 115° (polymorph *C*2_b), and again with a single protein molecule in the asymmetric unit. The protease dimer in this case is also created by the crystallographic twofold axis. Three other structures were also determined in the same space group, but they were non-isomorphous with the two major *C*2 crystal forms (two structures in polymorph *C*2_d and one in polymorph *C*2_c), the former with a dimer in the asymmetric unit and the latter with a dimer formed by crystal symmetry.

Other polymorphs included 11 structures in two non-isomorphous cells in the monoclinic space group *P*2_1_, seven structures in space group *P*2_1_2_1_2_1_, one structure in space group *P*2_1_2_1_2, three structures in space group *P*6_1_22, one structure in space group *P*3_2_21 and three structures in space group *P*1. The full biological dimer is present in the asymmetric unit of the crystals in space groups *P*2_1_2_1_2_1_ and *P*2_1_, whereas the functional dimers are created by crystal symmetry in space group *P*2_1_2_1_2 and by both noncrystallographic and crystallographic symmetry in the trigonal space group *P*3_2_21. The three non-isomorphous structures in space group *P*1 contain one or two dimers in the asymmetric unit. This large variety of crystal forms provides an excellent opportunity for investigating the potential influence of crystal contacts on protein structure in general and on the structure of 3CL^pro^ in particular.

Some of the structures required major changes to improve them in general, as well as to make their superposition more direct. In addition to the reindexing of the *I*2 data mentioned above, two structures, originally designated PDB entries 7jox and 7joy (Lee *et al.*, 2020 ▸), required special attention, since despite the similarity of their unit-cell parameters, which suggested isomorphism, they were not directly superposable. The reason was that the monoclinic β angles of these two crystals, as originally deposited in the PDB, were very close to and slightly above 90°, whereas it was necessary to make one of them slightly lower than 90° (β′ = 180° − β) and to reindex the diffraction data accordingly (−*h*, −*k*, *l*) in order to make the isomorphism obvious and the two structures directly compatible. This was communicated to the original authors of these depositions, who redeposited PDB entry 7jox in the PDB with the new settings (now designated PDB entry 7khp) and updated PDB entry 7joy by applying *ACHESYM*. The reindexing and re-refinement is also reflected in the covid-19 database.

All models that needed correction were re-refined with *REFMAC*5 (Murshudov *et al.*, 2011 ▸) and rebuilt with *Coot* (Emsley *et al.*, 2010 ▸). We utilized the structure-factor mtz or cif files downloaded from the PDB as data input. In many cases it was difficult to reproduce the original refinement statistics reported in the PDB files, especially if the refinement was originally performed with *Phenix* (Liebschner *et al.*, 2019 ▸) or *BUSTER* (Blanc *et al.*, 2004 ▸). In particular, the inconsistent way of reporting the ADPs when structures were refined with TLS parameters was a major obstacle in initiating new rounds of refinement. Another serious problem was the lack of definition of stereochemical restraints for many inhibitors. Such restraint files had to be recreated by us, but there was no guarantee that they would correspond exactly to the restraints used in the original refinements.

During our analysis, we noted many inconsistencies in the diffraction data deposited in the PDB. For example, the number of measured unique reflections for PDB entry 6wtj was 21 581, whereas the number reported to be used for refinement was 41 320. This discrepancy might possibly be due to the use of unmerged Bijvoet pairs during anomalous refinement, but there is nothing to indicate such a possibility in the REMARK section of the PDB file, and the structure is not described in a publication. In any case, the number of reflections in the structure-factor file used for map calculation agrees with the number measured, and these data were used by us in the re-refinement.

## 5. Analysis of the structures and assessment of their quality   

Our analysis will begin with a discussion of specific experimental conditions that might affect the final refined models, followed by a detailed dissection of structures of 3CL^pro^ determined in the presence of specific ligands. Finally, we will present a global comparison of all available models, also bringing the ligand-free (‘apo’) structures into the picture, in an effort to extract correlations with the crystallization of the enzyme in different polymorphs and with other experimental and structural effects.

### 5.1. Low- versus room-temperature structures   

**Figure 3:**
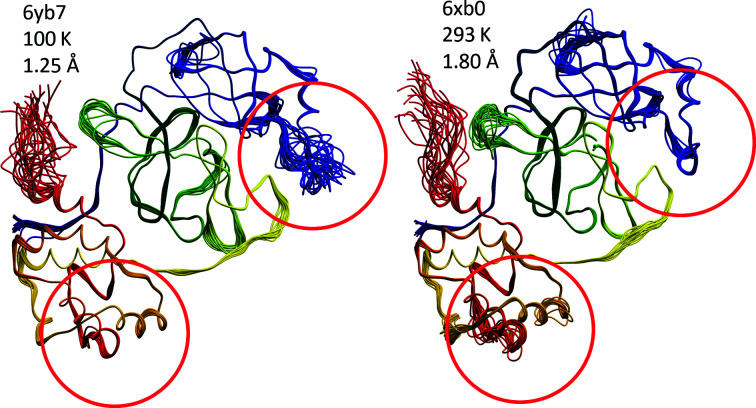
Local conformational differences between low-temperature and room-temperature (RT) structures of 3CL^pro^ *C*2 polymorphs. Shown in the same orientation as in Fig. 1 ▸ are 25 multiconformer refinement molecular-dynamics (MD) traces of one protomer of each crystallographically symmetric dimer in the most frequent polymorph *C*2_a (PDB entry 6yb7, cryogenic temperature; PDB entry 6xb0, room temperature). The low-temperature model with PDB code 6yb7 has lower overall *B* factors (thinner trace bundles) and a corresponding higher resolution, but the MD traces show that local differences in conformational variability can be significant. The circled region at the top right of the molecule in the low-temperature model with PDB code 6yb7 exhibits flexibility similar to the cryogenic model with PDB code 7jkv shown in Fig. 1 ▸(*b*), while the RT model seems to be less variable in this region. In contrast, the helical region (bottom, red) seems to be less ordered in the RT model. At the same time, the overall low r.m.s.d.s between models remain comparable to the DPI estimates and do not inform about large local variances.

Thirteen models of the 3CL^pro^ structure in three polymorphs of both the free and inhibited enzyme were refined using diffraction data collected at room temperature (RT), allowing the old question of whether structures determined at liquid-nitrogen temperatures differ from those determined at the physiologically more relevant room temperature to be addressed. The diffraction data were collected by several research groups using different approaches. A rotating-anode generator equipped with a large pixel detector was used to collect data for the free enzyme (PDB entries 6wqf, 6xb0 and 6xb1; Fig. 3 ▸) and for several inhibitor complexes (PDB entries 6xb2, 6xqs, 6xqt, 6xqu and 6xch). X-ray diffraction data collected in the same way were also used in a joint refinement with neutron diffraction data (PDB entry 7jun). Finally, X-ray free-electron laser (XFEL) data were utilized to refine three crystal structures of the uninhibited protein (PDB entries 7cwb, 7cwc and 7jvz).

A detailed analysis of the 2.3 Å resolution structure of the uninhibited enzyme obtained at room temperature (PDB entry 6wqf) is available (Kneller, Phillips, O’Neill, Jedrzejczak *et al.*, 2020 ▸). The authors suggest that the ‘room-temperature structure of the 3CL M^pro^ ligand-free form may be the more physiologically relevant structure for performing molecular-docking studies to estimate drug binding and enable drug design’. They provided at least two different examples showing that the room-temperature structure differs from its counterpart at low temperature. They noted that the ‘catalytic water’ molecule, usually located between His41, His164 and Asp187, is not present in the 2 Å resolution structure obtained from flash-cooled crystals (PDB entry 6m03). However, although this crucial water molecule was not modeled in the coordinate set deposited with PDB entry 6m03, it is very clearly present in the electron-density map and its omission is an obvious error. This conclusion is also supported by all other ligand-free 3CL^pro^ structures obtained at cryogenic temperature, in which the corresponding water molecule is clearly visible in the electron density. The other reported difference between the room-temperature and low-temperature structures involves a different conformation of residues 192–198, with the peptide bond of Ala194 in PDB entry 6wqf flipped compared with the 100 K structure (PDB entry 6y2e). While supported by the electron-density maps, the purported importance of this feature is undercut by the fact that other room-temperature structures, including that resulting from the joint X-ray/neutron refinement by the same team (Kneller, Phillips, Weiss *et al.*, 2020 ▸; PDB entry 7jun), do not show a similar peptide flip.

The three structures of the free enzyme obtained with XFEL radiation were determined using crystals in two space groups: *P*2_1_2_1_2_1_ at 2.1 Å resolution (PDB entry 7cwc) and two almost isomorphous structures in space group *C*2 reported at 1.9 Å resolution (PDB entry 7cwb) and 2.5 Å resolution (PDB entry 7jvz). In the absence of data-scaling statistics for the XFEL structures it is not possible to assess the quality of the structure amplitudes used in their refinement. The latter two structures are also almost isomorphous with some of the structures, including the reindexed PDB entries 6xb0 and 6xb1 obtained using a traditional experimental approach that were discussed above. The two isomorphous structures (PDB entries 7cwb and 7jvz) superpose with an r.m.s.d. of 0.67 Å for all 306 C^α^ atoms, whereas superposition of PDB entry 7cwc (chain *A*) onto PDB entry 7cwb results in an r.m.s.d. of 1.2 Å for 297 C^α^ atoms. The conformation of the 192–198 fragment is consistent in PDB entries 7cwb and 7cwc and is the same as in the room-temperature structure PDB entry 6wqf, but this peptide has the same orientation in PDB entry 7jvz as in the 100 K structure with PDB code 6y2e. Superposition of PDB entry 6wqf on PDB entry 7cwb results in an r.m.s.d. of 0.42 Å, which is lower than even that for the two isomorphous XFEL structures. These comparisons clearly demonstrate that structural changes related to temperature are not as large as expected, and are easily masked by many other factors, for instance crystal packing.

**Figure 4:**
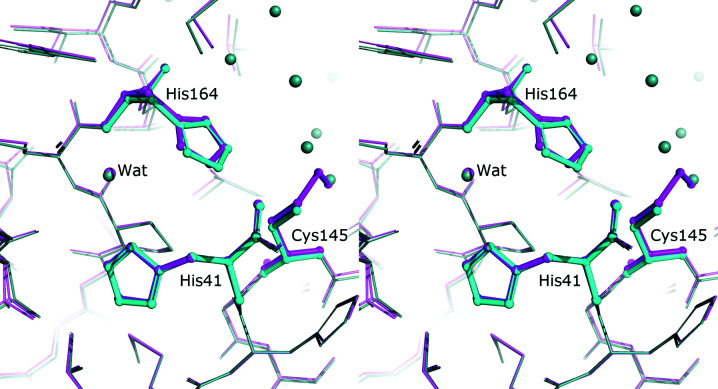
Stereo figure showing a superposition of the coordinates of the active sites of the highest resolution 3CL^pro^ structures determined at room temperature (PDB entry 6xb0; magenta) and at 100 K (PDB entry 6yb7; cyan). The superposition is based on all C^α^ atoms. The catalytic water molecule is labeled Wat.

A superposition of the coordinates of the active sites of the 3CL^pro^ structures for the highest resolution data sets obtained at room temperature (PDB entry 6xb0) and at 100 K (PDB entry 6yb7) is shown in Fig. 4 ▸. It confirms that the differences between structures determined with data collected at different temperatures and using different kinds of radiation sources are not significant (r.m.s.d. of 0.38 Å for all 306 C^α^ pairs), despite the oxidation of the catalytic cysteine in the room-temperature structure. Structures determined with radiation generated by a conventional X-ray source and by an XFEL are also not significantly different, with the differences between XFEL structures belonging to different space groups vastly exceeding the differences due to either temperature or radiation source. These results may lead to a generally applicable conclusion that, in the absence of special conditions that would clearly require data collection at room temperature or using very short pulses of radiation, crystallographic data obtained at standard cryogenic temperatures may be sufficient for the interpretation of the details of the active sites of enzymes required to propose ligand binding and ultimately for drug design. This is at least clearly the case for 3CL^pro^.

### 5.2. Complexes of 3CL^pro^ with ligands   

**Figure 5:**
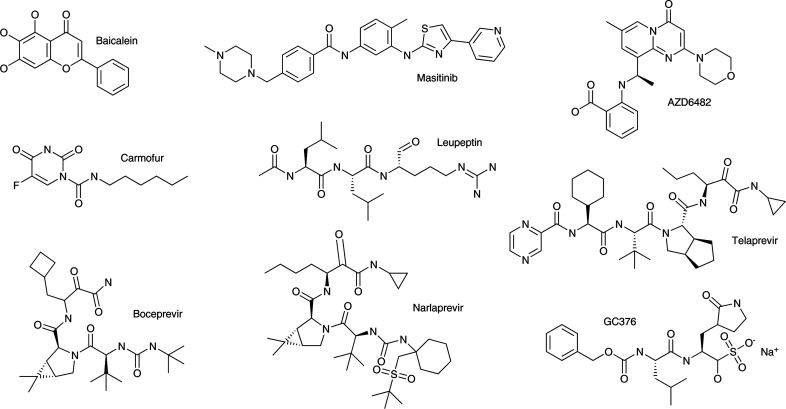
Chemical formulas of selected inhibitors of 3CL^pro^ discussed in this paper. The inhibitors are baicalein, masitinib, AZD6482, carmofur, leupeptin, telaprevir, boceprevir, narlaprevir and GC376.

The majority of the 57 3CL^pro^ complexes analyzed here utilized compounds that have been characterized in the past because of their ability to inhibit virally encoded proteases, particularly SARS-CoV 3CL^pro^. Most of the inhibitors are dipeptide analogs containing amino-acid residues with highly unusual side chains, equipped at the N- and C-termini with various chemical moieties (Fig. 5 ▸). Only seven non-oligopeptidic small-molecule compounds are found among those complexes, including one with only a single peptide bond. Almost all inhibitors are covalently bound to the catalytic Cys145, with only three structures containing noncovalently bound inhibitors (Supplementary Table S1). Multiple structures are available for some of these inhibitor complexes, whereas other inhibitor structures were determined only once or twice. Here, we will discuss the latter structures first, with those seen more frequently discussed together later.

#### 5.2.1. Structures with noncovalently bound inhibitors   

Only three structures of complexes with noncovalently bound inhibitors are present amongst those that have been analyzed by us. A complex of 3CL^pro^ with baicalein, a component of the traditional Chinese herbal medicine Shuanghuanglian, was described by Su and coworkers (PDB entry 6m2n; Su *et al.*, 2020 ▸). The structure in space group *P*1 includes four protein molecules in the asymmetric unit, with the inhibitor clearly visible in each of them. Electron density for the fused double-ring structure is clearly seen in all of the molecules, whereas the terminal phenyl group is poorly ordered in two of them and lacks support from the electron density. The compound is bound in the direct proximity of Cys145 and is held in place by several hydrogen bonds to the main-chain amides of the enzyme. A stacking interaction with the imidazole ring of His41 anchors the inhibitor without displacement of this residue.

The inhibitor masitinib is bound noncovalently in PDB entry 7ju7 with part of it close to Cys145 (the distance between the S^γ^ atom of Cys145 and the N atom of the thiazole ring of the inhibitor is only 3.3 Å). This bulky compound is largely located on the surface of the enzyme and there is practically no electron density for almost half of the molecule.

Binding of AZD6482 was noticed in a search for potential allosteric inhibitors of 3CL^pro^ conducted through a massive X-ray screen of two repurposing drug libraries (Günther *et al.*, 2020 ▸). The compound binds on the surface of the catalytic domain away from the active site, and also far from the dimer interface, and is wedged by a symmetry-related protein molecule that is not part of the same protease dimer. The aminobenzoate moiety is adjacent to His80, Lys88 and Lys90, while most of the remainder of the inhibitor sits on the surface of the enzyme, with only N^δ2^ of Asn63 forming a hydrogen bond to the keto group in the pyrimidine ring of the inhibitor. Extensive interactions with the symmetry-related molecule raise doubt as to whether the mode of binding in this crystal structure could correspond to any authentic mode of binding of this compound in solution.

#### 5.2.2. Covalent inhibitors seen in a limited number of structures   

All other peptidic and nonpeptidic inhibitors are covalently linked to the S^γ^ atom of the active-site Cys145 residue of the protease. The next three nonpeptidic inhibitors contain heterocyclic ring moieties, including succinimide (designated NEN in PDB entries 6xb1 and 6xb2), indazole (designated GKF in PDB entry 7cx9) or a pyridine conjugated with a ring containing oxygen, sulfur and zinc (designated PK8 in PDB entry 6yt8). The fourth compound, carmofur (hexylcarbamic acid; acronym JRY), is complexed in PDB entry 7buy.

The succinamide ring C atom of NEN in PDB entries 6xb1 and 6xb2 forms a direct covalent bond to S^γ^ of Cys145, while the carmofur moiety forms a flat thioester connection through its carboxyl group. It is not clear how the PK8 compound is attached to the enzyme in PDB entry 6yt8, since the electron-density map does not support the original modeling with two disordered molecules. PDB entry 7cx9 with inhibitor INZ-1 (designated in the PDB as GKF) is one of the two covalent complexes in which the value of the torsion angle N—C^α^—C^β^—S^γ^ in Cys145 (−155°) is far outside the range found in all other covalent complexes (−50° to −95°) (Supplementary Table S2). The compound INZ-1 in the model with PDB code 7cx9 forms a flat thioester connection, although it is presented as an aldehyde in the PDB. This case illustrates a notorious problem in the PDB of inappropriate differentiation between S^γ^ linkages with carboxylic versus aldehyde/ketone groups of the inhibitors. In the former moiety the connecting thioester group is flat, whereas in the latter case the connecting thiohemiacetal (or thiohemiketal) group contains a chiral *sp* ^3^ C atom with either *R* or *S* chirality (and sometimes both). In the description of many complexes shown in Table 2 ▸ (and in other structures in the PDB as well) the proper nomenclature is not used.

All remaining inhibitors in Table 2 ▸ are oligopeptide analogs. That with the most standard residues is leupeptin, Ace-Leu-Leu-argininal, which is present in PDB entries 6xch and 6yz6. The only unusual part of this inhibitor is the argininal moiety, *i.e.* an aldehyde version of arginine. The complex of 3CL^pro^ with leupeptin is discussed separately below. Most residues and other moieties present in all of the other peptidic inhibitors are not encountered in natural proteins.

The covalent peptidic inhibitor that does not form an acetal/ketal or ester bond with the S^γ^ atom of Cys145 is N3, which is present in two structures: PDB entries 7bqy and 6lu7. This dipeptidic compound contains a C=C double bond and the complex is a simple covalent adduct of the SH group of Cys145 to this double bond.

There are four structures where the carboxylic group of the inhibitor forms a covalent link to Cys145, in which the connecting C atom is *sp* ^2^ hybridized and the entire thioester group is flat. Apart from the above-mentioned structures PDB entries 7buy and 7cx9 (with the small-molecule inhibitors carmofur and INZ-1, respectively), the peptidic structure in PDB entry 7c8t forms the same planar connection with the inhibitor TG0205221 (designated NOL in the PDB). The fourth example of a planar connection with Cys145 is the acyl-enzyme intermediate structure PDB entry 7khp, in which one enzyme molecule forms a covalent thioester product with its symmetry mate. This structure is analyzed in detail below.

All of the other complexes in Table 2 ▸ are formed by the reaction of aldehyde or ketone groups of the inhibitors with the thiol group of Cys145, leading to the formation of hemithioacetal or hemithioketal linkages, respectively. Among the 38 coordinate sets, there are 23 hemiacetals and 29 hemiketals, with some structures containing multiple molecules. In all of these complexes the C atom connected to the cysteine S^γ^ atom is chiral due to *sp* ^3^ hybridization and has a hydroxyl OH group as one of its substituents. A characteristic difference between the hemiacetal versus hemiketal connections is that the OH group of hemiacetals is typically directed towards the oxy­anion hole, which consists of the main-chain amides of residues 143–145, and forms a hydrogen bond to the peptide N atom of Cys145, whereas in hemiketals this group points in the opposite direction and forms a hydrogen bond to the N^ɛ2^ atom of His41. In both cases the configuration of the asymmetric C atom remains *S*, even though the direction of the OH group is opposite. This confusing nomenclature is due to the change of substituent priority at the linking C atom. There are only a few exceptions to this rule. The first case comprises two hemiacetal-containing structures (PDB entry 6yz7 and molecules *A* and *B* of PDB entry 7d1m) which display disorder of this group, with partially occupied hydroxyl O atoms in both positions. A second exception is the hemiketal-containing complex with UAW243 in PDB entry 6xfn with *R* chirality and with the OH group hydrogen-bonded to the N^ɛ2^ atom of His41. Moreover, the hemiacetal connection in molecule *C* of PDB entry 6wtt is presented in the PDB with *R* chirality of the central C atom and with the hydroxyl group hydrogen-bonded to N^ɛ2^ of His41, contrary to the evidence from the difference electron density and subsequent re-refinement, which clearly supports the standard *S* configuration as in the two other molecules in this structure. The structure with PDB code 6xfn is the other case in which the χ_1_ torsion angle N—C^α^—C^β^—S^γ^ of Cys145 has an unusual value of about +35°, whereas in all other structures with a chiral C linker atom this angle lies in the range between −50 and −95°.

#### 5.2.3. Leupeptin   

Leupeptin is a well known tripeptide inhibitor of serine and cysteine proteases. The two structures of its complex with 3CL^pro^ represent an interesting case of different approaches to modeling the inhibitor molecule and its link to the active-site residue, rather than genuine differences. The structures in question were determined at 2.2 Å resolution at room temperature (PDB entry 6xch; Kneller, Galanie *et al.*, 2020 ▸) and at 1.7 Å resolution under cryogenic conditions (Fig. 3 ▸; PDB entry 6yz6; Günther *et al.*, 2020 ▸). The description of the inhibitor and its link to Cys145 is conventional in the room-temperature structure, with only a single *R* stereoisomer of the link present in the model and a distance between Cys145 S^γ^ and C4 of the inhibitor of 1.8 Å, with no indication of the presence of the second stereoisomer. However, in the low-temperature structure there is a very clear indication of the presence of both diastereomers, which evidently resulted from the nonstereospecific character of the inhibition reaction. To take account of this situation, the original authors of PDB entry 6yz6 modeled the inhibitor as two overlapping leupeptin molecules differing only in the absolute configuration of the substituents at the C4 atom. Despite the 1.7 Å resolution of the diffraction data, such modeling resulted in an unlikely S^γ^–C4 distance of only 1.4 Å. When the structure was re-refined by us with a single leupeptin molecule and with the O4 atom of the argininal (AR7) moiety assumed to be in two alternative configurations, the model fitted the electron-density map much better and the S^γ^–C4 distance converged at 1.8 Å. These two structures illustrate how nonparsimonious modeling and contradiction of established rules can result in implausible models, leading to spurious differences that do not represent any real variations between structures.

#### 5.2.4. Telaprevir   

Telaprevir (VX-950) is an FDA-approved drug that was originally designed and characterized by Vertex Pharmaceuticals as an inhibitor of the NS3-4A serine protease of HCV (Lin *et al.*, 2006 ▸). The compound was derived from the NS5A/5B viral substrate of the protease using structure-based drug-design techniques and was found to be a covalent (albeit reversible) inhibitor of the enzyme. Despite the differences in the catalytic nucleophile and specificity of the HCV NS3-4A and SARS-CoV-2 3CL^pro^ proteases, telaprevir has been investigated as a potential drug candidate for COVID-19, including the determination of crystal structures of its complexes with the 3CL^pro^ enzyme. Four structures are isomorphous in space group *C*2 with a single protein molecule in the asymmetric unit, whereas the fifth structure (PDB entry 7c7p) is in space group *P*2_1_2_1_2_1_ with a dimer in the asymmetric unit (Table 2 ▸). PDB entry 6xqs was determined at room temperature with a home X-ray source, whereas the data for the other crystals were collected using synchrotron radiation at cryogenic temperature.

The electron density for the inhibitor is unambiguous, with the exception of the partially disordered inhibitor bound to chain *B* in the orthorhombic structure with PDB code 7c7p. Strangely, without any logical justification, this partially modeled inhibitor was labeled FK3 by the PDB, whereas enzyme-bound telaprevir is labeled SV6. In all structures the hydroxyl group in the covalent linkage between the S^γ^ atom of Cys145 and the inhibitor forms a very short hydrogen bond to N^ɛ2^ of His41, which is part of the catalytic dyad. This interaction provides additional stabilization of His41, which in turn interacts with the catalytic water through a hydrogen bond involving N^δ1^. The conformation of the norvaline side chain is somewhat variable in the absence of good hydrophobic interactions, and the end of the inhibitor chain is not visible in the electron-density maps in any of these structures. A large, unexplained difference density is seen adjacent to the nor­valine residue in the structure with PDB code 6zrt. The structures of the cyclopentane-coupled proline and *tert*-butyl side chains are very similar in all complexes, although some chiral C atoms appear to have a visibly planar character enforced by the erroneous restraints used for the model in PDB entry 6xqs. The conformation of the cyclohexyl side chain varies between the structures, with the chirality of the CBH atom differing from the other three structures in PDB entry 6zrt and in molecule *A* of PDB entry 7c7p. It is very likely that this difference is not real, but rather reflects different (not always correct) stereochemical restraints in the refinement of the individual structures. Finally, the terminal pyrazine moiety is virtually identical in the monoclinic structures due to its stabilization by crystal contacts, but diverges very significantly in molecule *A* of the orthorhombic structure, where it is adjacent to the ring of Pro168. This part of the molecule is not observed in molecule *B*.

#### 5.2.5. Boceprevir   

Similarly to telaprevir, boceprevir (SCH503034) is an FDA-approved drug that was originally designed and characterized by Schering–Plough as an inhibitor of the NS3-4A serine protease of HCV (Prongay *et al.*, 2007 ▸). Boceprevir was later brought into clinical practice by Merck as a hepatitis C drug, although it is no longer used for this purpose. Its structure was first determined in complex with the HCV protease (Prongay *et al.*, 2007 ▸) and it was one of the first previously characterized protease inhibitors that was shown to be a potent inhibitor of SARS-CoV-2 3CL^pro^ (Ma *et al.*, 2020 ▸). Seven structures of complexes of boceprevir with 3CL^pro^ were deposited in the PDB during the time frame of this analysis (Table 2 ▸), providing a total of nine crystallographically independent views. Their resolution ranges from 2.25 to 1.35 Å and, except for the room-temperature structure with PDB code 6xqu, the diffraction data were collected using synchrotron sources at cryogenic temperature.

It should also be noted that the description of boceprevir in the PDB is confusing. The compound is named HU5 as the unreacted drug and U5G as the fragment of the molecule that results from chemical reaction with the enzyme. One could argue that either way of defining the group has some merit, but since boceprevir is covalently bound to the catalytic serine or cysteine in all structures, its description as the unbound drug confuses not only users but even the graphics program *Coot* (Emsley *et al.*, 2010 ▸), which displays the hydroxyl O atom O33 of the hemithioketal group with a double bond. In our re-refinement of all structures of these 3CL^pro^ complexes, we have consistently identified the bound form of boceprevir as U5G.

The structures with PDB codes 6xqu and 6zru were originally determined and deposited in the nonstandard space group *I*2, but we reindexed the data and placed the models in the equivalent, standard space group *C*2 to make comparisons with the isomorphous *C*2_a structures easier. PDB entries 7c6s and 6wnp belong to polymorph *C*2_b and show different crystal packing compared with the other *C*2 complexes with boceprevir. The remaining two boceprevir complex structures belong to space groups *P*2_1__b (PDB entry 7brp) and *P*2_1_2_1_2_1_ (PDB entry 7com), with a 3CL^pro^ dimer in each asymmetric unit.

In all structures the electron density is unambiguous and supports the positioning of the ligand well. The conformation of the inhibitor is practically identical in all structures. Only the cyclobutyl group seems to be disordered in PDB entry 7k40, the highest resolution structure. Analogously to the structures with telaprevir, the N^ɛ2^ atom of His41 is involved in a short hydrogen bond to the hydroxyl group of the hemithioacetal in the inhibitor–enzyme covalent link. The molecule is further stabilized by three N—H⋯O hydrogen bonds from Gly143, Ser144 and Cys145 to the O atom of the amide group adjacent to the covalent linker. Moreover, the inhibitor molecule also creates a set of β-sheet-like hydrogen bonds with His164 and Glu166.

#### 5.2.6. Narlaprevir   

Narlaprevir (SCH 900518, Arlansa) is currently approved for clinical use in Russia as an inhibitor of the NS3-4A serine protease of HCV. Previously, its structure was investigated in complex with the NS3-4A protease of HCV (PDB entry 3lon, unpublished work). Three structures of the complexes of narlaprevir with SARS-CoV-2 3CL^pro^ were deposited in the PDB during the time period of this study: two of them are isomorphous in space group *C*2_a with a single protein molecule in the asymmetric unit, with data collected at cryogenic temperature (PDB entries 7d1o and 7jyc), whereas the third (PDB entry 6xqu) is a room-temperature structure in space group *P*2_1_ with one dimer in the asymmetric unit (Table 2 ▸, Supplementary Table S1).

Superposition of the two isomorphous structures yields an r.m.s.d. of 0.46 Å for 301 C^α^ pairs, whereas superposition of either chain *A* or chain *B* of PDB entry 6xqu on PDB entry 7jyc yields an r.m.s.d. of 0.49 Å, indicating that the differences in crystal packing or temperature of the experiment did not result in any significant changes in the protein model. The electron density for the inhibitor in all four complexes is unambiguous, showing the C atom covalently bound to S^γ^ of Cys145 (C43) in the *S* configuration. The hydroxyl group bound to C43 is within hydrogen-bonding distance of the side chain of His41, the second residue of the catalytic dyad. No other configuration of C43 is possible due to the presence of a bulky cyclopropylcarbamoyl group that replaced an H atom. The whole inhibitor molecule has practically the same conformation in each complex, and narlaprevir also assumes the same conformation in complex with the HCV protease, despite large differences between the two enzymes in the vicinity of the active site. The three amide N atoms of narlapravir form hydrogen bonds to the main-chain carbonyl groups of His164 and Glu166, whereas one of the carbonyl O atoms of the inhibitor is the acceptor of a hydrogen bond from the amide N atom of Glu166, again zipping an inhibitor–enzyme β-sheet motif. The terminal *tert*-butylsulfonyl group is not involved in any direct interactions with the protein.

It should be noted that the reference compound for narlaprevir in the PDB (defined as NNA) does not correspond to the parent drug, but rather to the part of it that is found in the complex with a protease. The configuration of C43 is marked as *R*, but this is due to the assumption that one of the atoms bound to it is an H atom and not a heavy substituent attached via an S atom. The absolute configuration of the C43 chiral center listed in the PDB should therefore be treated as dubious, at least.

#### 5.2.7. GC376 and its variants   

GC376 is a prodrug of a dipeptide inhibitor that contains a sulfonic group warhead that is cleaved off when the compound reacts with the active-site nucleophile of a protease, forming a covalent adduct, which in the case of 3CL^pro^ is a thiohemiacetal link at the thiol group of the catalytic Cys145 residue. The inhibitor was designed as a member of a series of compounds that had generally similar structures, but different reactive groups, and that were shown to be active against a variety of viral proteases, including those of coronaviruses (Kim *et al.*, 2012 ▸). Seven structures of complexes of 3CL^pro^ with GC376 have been analyzed in this project. They were all obtained under cryogenic conditions and their resolutions range from 2.35 to 1.35 Å. The unreacted parent prodrug was assigned the code K36 by the PDB, with an *S* configuration at the C21 reactive center. In the complexes, the sulfonic group of K36 is substituted by the S atom of Cys145, with the chiral C21 atom of the inhibitor again linked to sulfur. The configuration at C21 of the conjugated K36 inhibitor is marked as *S*, but a virtually identical conjugate, named B1S, in which the chirality at the C21 center is *R*, was included in the structure with PDB code 6wtt. In light of the robust and unique orientation of the bulk of the inhibitor attached to C21, inversion of the configuration of this atom consists of swapping the sulfur substituent and the OH group of the hemiacetal group. In the structure with PDB code 6wtt there are three complex molecules in the asymmetric unit. In two of them the ligand was defined as K36, while in the third it is B1S. To add to the confusion, an inhibitor identical to B1S was defined as UED in the structure with PDB code 6wtk. An attempt to clarify the situation by removal of the unnecessary multiplication of standard PDB groups was further complicated by the use of a vastly different numbering of identical atoms, a problem that has been identified in the PDB in the past (Jaskolski, 2013 ▸) but still not ameliorated. After re-refinement of PDB entry 6wtt we concluded that, in any case, the chirality at C21 was identical in all three copies of the complex present in the asymmetric unit.

Analysis of the seven structures of the GC376 complex indicated a number of problems in some of them. The structure with PDB code 7cbt was a particular outlier, since part of the main chain of molecule *B* (extending between residues 211 and 290) had signs of serious mistracing, which is rather surprising in a structure determined by molecular replacement using the structure with PDB code 6y2e, which did not suffer from such problems, as a model. Several other parts of the model also had to be corrected during re-refinement. An inconsistency in the deposited diffraction data was found for PDB entry 6wtk. This structure was originally refined at 2 Å resolution, although data apparently extending to 1.2 Å resolution were present in the structure-factor file, indicating an attempt to measure reflections far beyond the real diffraction limit. Our re-refined model utilized the original 2 Å resolution data. In collaboration with the original depositors of PDB entry 7brr, we reprocessed the diffraction data to 1.4 Å resolution, re-refined the structure and redeposited it as PDB entry 7d1m, replacing the original entry. Due to reprocessing and the use of fully anisotropic ADP parameters, the resulting electron-density maps improved sufficiently to allow us to add an alternative conformation to the bound ligand and to correct the conformations of a number of side chains.

**Figure 6:**
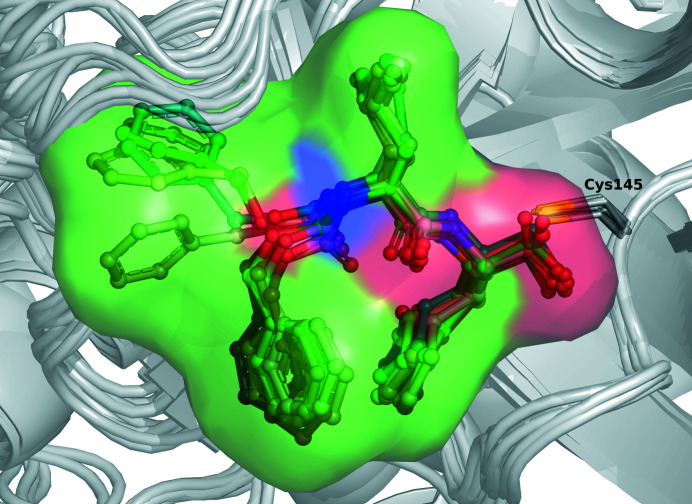
A comparison of the 11 independent views of the pose of GC376 bound to 3CL^pro^. Superposed inhibitor molecules in seven structures are shown, PDB entries 6wtj, 6wtk, 6wtt (chains *A*, *B* and *C*), 7c6u, 7c8u, 7cbt (chains *A* and *B*) and 7d1m (chains *A* and *B*), with chain *A* of 7d1m serving as a reference. The GC376 inhibitors are shown in ball-and-stick representation with C atoms in green, O atoms in red and N atoms in blue. The surface represents all 11 overlapped ligands in their respective atomic colors. The protease backbone is shown in gray and the side chain of the catalytic Cys145, at the inhibitor–enzyme covalent link, is labeled.

Molecule *A* of PDB entry 7d1m was used as a reference for comparisons of this series of structures, which yielded 11 independent views of the mode of inhibitor binding (Fig. 6 ▸). In comparison with the other structures obtained at cryogenic temperature, the r.m.s.d. between superposed C^α^ atoms was 0.68–0.75 Å for 298–299 C^α^ pairs. The only possible exception was noted during comparison with the structure with PDB code 6wtt obtained from trigonal crystals, in which each of the three molecules in the asymmetric unit yielded an r.m.s.d. of 0.92–0.99 Å from the reference molecule. A comparison with the isomorphous structure with PDB code 7cbt yielded r.m.s.d.s of 0.50 Å for chains *A*, 0.54 Å for chains *B* and 0.57 Å for an overall superposition of both chains. The divergence between molecules *A* and *B* in the same crystal structure, PDB entry 7d1m, is considerably larger, 1.53 Å for 298 C^α^ pairs, with significant deviations found in particular in the stretch of residues 222–227. It is clear that crystal contacts were responsible for these much more significant differences.

Although nominally a dipeptide, the side chains of GC376 can fill three substrate-binding subsites, S1–S3, of the enzyme. The covalent link between Cys145 and the inhibitor indicates that the reaction leading to the departure of the sulfonic group is not completely stereospecific, since in at least two structures (PDB entries 7d1m and 7c8u) both the *R* and *S* diastereomers of the thiohemiacetal atom C21 can be unambiguously discerned in the electron density. Only the predominant *S* isomer was modeled in the other GC376 complex structures, with the hydroxyl O22 atom pointing into the oxyanion hole of the enzyme. O atom O30 of the 2-pyrrolidone group that occupies the S1 subsite of the enzyme is the acceptor of a very strong hydrogen bond from the N^ɛ2^ atom of His164 and possibly also from N^δ1^ of His172. However, the conformation of the latter residue is inconsistent among the compared structures, since its potential interactions with the main-chain carbonyls of Ser1 and Gly138 (or Ile136) may lead to different interpretations of the hydrogen-bond network. Additional stabilization of the P1 residue of the inhibitor is provided by another strong hydrogen bond between the amide N atom of the lactam group and O^ɛ2^ of Glu166.

The side chain of the P2 Leu of the inhibitor is wedged in the largely hydrophobic S2 pocket of the enzyme and its conformation is virtually identical in all compared structures. On the other hand, the terminal P3 benzyl group, which was expected to fill the S3 pocket, points in various directions in some of the structures. The electron density is unclear or even absent in some of the models, suggesting that this group might have been hydrolyzed during the course of the crystallization experiment.

Five structures of complexes of 3CL^pro^ with inhibitors structurally related to GC376 (PDB entries 6xa4, 6xfn, 6xbg, 6xbh and 6xbi) were published by Sacco *et al.* (2020 ▸). The compounds are named UAW241, UAW243, UAW246, UAW247 and UAW248, respectively, in the PDB depositions, whereas the first two are named calpain inhibitor II and calpain inhibitor XII in the publication, with the other three named there as UAWJ24*x*. Re-refinement of PDB entry 6xfn uncovered incorrect chirality of the P1 norvaline residue in the original model, which is most likely a consequence of incorrect restraints for this moiety. This problem was corrected during the re-refinement. The binding mode of UAW243 is very different from the other inhibitors in this series, in that the nominally P1 norvaline occupies the S1′ pocket of the enzyme, whereas the P1′ pyridine occupies the S1 pocket. The P2 leucine and P3 cyclohexyl residues point out into the solvent, with the very weak electron density of the latter residue raising the question of whether it is still actually present or whether it has been hydrolyzed during the crystallization experiment. The conformation of the other four inhibitors is internally very consistent, as well as consistent with GC376, although the formal chirality of the C21 atom (in GC376 nomenclature) depends on the presence or absence of the P1′ side chain of the inhibitor. In all structures the P1 side chain is stabilized by a hydrogen bond to N^ɛ2^ of His164. The hydrophobic P2 residue (Leu or Phe) occupies a pocket with mixed hydrophobic/hydrophilic character, and the (also hydrophobic) P3 residues, which are located on the surface of the enzyme, assume a highly variable conformation.

### 5.3. Structures with direct relevance to the analysis of substrate binding and the mechanism of action   

Two structures are of particular interest for the analysis of the substrate-binding mode and activity of the enzyme, namely that of the acyl-enzyme intermediate of the reaction (PDB entry 7khp) and of a product complex (PDB entry 7joy) (Lee *et al.*, 2020 ▸). These structures resulted from a remarkably serendipitous crystallization of the native and C145A variants of 3CL^pro^: while molecules *A* of the dimer in the asymmetric unit were unremarkable, molecules *B* were found to form an infinite chain in the crystal by inserting the C-terminus of one molecule into the active site of its translational copy. This packing arrangement was identical in both structures, but whereas an unmodified C-terminus was observed in the crystals of the C145A active-site mutant, in the catalytic site of the wild-type enzyme the wedged carboxylate of Gln306 was found to be linked via a covalent acyl-enzyme bond to the Cys145 nucleophile. The binding of the carboxylic group illustrates the reversible character of the reaction catalyzed by 3CL^pro^. In the forward direction, a peptide bond is split into its amino and carboxy constituents. In the reverse direction observed in PDB entry 7khp, the carboxy substrate forms an acyl-enzyme thio­ester link at Cys145 but the reaction has to stop there as there is no amino half-substrate/product for its completion. Although the above two structures were initially inconsistently presented, they were later modified by the original authors to emphasize their isomorphism (this required the replacement of PDB entry 7jox by PDB entry 7khp in the PDB and a shift of the coordinates of PDB entry 7joy).

**Figure 7:**
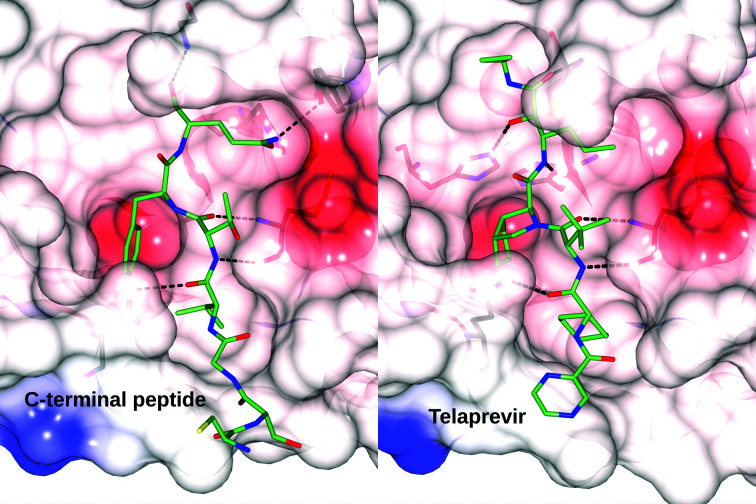
Side-by-side comparison of the binding of the autoprocessed C-terminal peptide (PDB entry 7khp) and telaprevir (PDB entry 7k6d) in the active site of 3CL^pro^. Hydrogen bonds are shown as dashed lines. The protein molecule is represented as a semi-transparent charge-density surface with positive charge shown in blue, negative charge in red and hydrophobic character shown in white. The ligands in the active site are shown in stick representation with C atoms in light green, O atoms in red and N atoms in blue. 3CL^pro^ residues forming hydrogen bonds to the ligands are shown as sticks under the charge-density surface.

A comparison of the acyl-enzyme complex structure with PDB code 7khp with the highest resolution structure of a complex with a long inhibitor molecule (telaprevir; PDB entry 7k6d) shows a remarkable similarity in the location of the side chains of the substrate and inhibitor in the S1–S4 pockets of the enzyme (Fig. 7 ▸). The presence of the covalent acyl-enzyme linkage in the inhibitor complexes did not lead to any significant rearrangement of the active-site residues compared with the free enzyme analyzed in the neutron study. In particular, the conformation of Cys145, the orientation of His41 and His164 and the location of the catalytic water molecule were highly conserved. The most significant shift of the main and side chains of the enzyme is due to the much larger P4 residue in the inhibitor, a cyclopentane-coupled proline, compared with the Val303 side chain of the substrate, culminating in repulsions exerted on Gln192 and also leading to a peptide flip of this residue. A notable feature of the structure of the acyl-enzyme intermediate in PDB entry 7khp is the presence of a weak electron-density peak located 2.66 Å from the C atom of Gln306 and 2.85 Å from the N^ɛ2^ atom of His41. This density was interpreted by Lee and coworkers as a water molecule poised for a nucleophilic attack on the thioester group. Since the protonation of these residues can only be inferred indirectly by modeling, a confirming neutron diffraction structure of this particular crystal form of 3CL^pro^ might be of particularly high interest.

Another structure (PDB entry 7jun) with direct relevance to the mechanism of action of 3CL^pro^ resulted from a joint X-ray/neutron refinement of the uninhibited enzyme at 2.3/2.5 Å resolution (Kneller, Phillips, Weiss *et al.*, 2020 ▸). This structure was not revised in our project since we did not have access to the required tools. For this reason, the analysis presented below is based on the nuclear density map kindly provided by the original authors. Parenthetically, we note that such a map is not directly available from the PDB.

Based on the appearance of the nuclear density map, Kneller and coworkers proposed a model in which both His41 and His164 were doubly protonated (cationic), while Cys145 was deprotonated (anionic). The question of the protonation state of these residues had previously been analyzed for other 3CL^pro^ enzymes using molecular dynamics by Paasche *et al.* (2014 ▸), who arrived at the conclusion that the most likely resting state consists of Cys145 and both histidines being neutral, while the zwitterionic state was proposed to consist of charged Cys145 and His41 with a neutral His164. At variance with this proposal, the putative protonation state of the neutron-based model assumes that both histidines are charged at the nominal crystallization pH of 6.6. This interpretation, however, would require the catalytic Wat409 to be the acceptor of three hydrogen bonds (from the N^δ1^ atoms of the two histidine residues and from the main-chain amide of His41), while both D atoms of this heavy water molecule would form deuterium bonds to O^δ2^ of Asp187. Such an arrangement is rather unlikely and considering that the nuclear density for Wat409 is quite featureless, whereas the density for D^δ1^ of His41 is lower than that for D^ɛ2^, one could postulate that Wat409 could be rotated. In the new arrangement this D_2_O molecule would become a hydrogen donor to both Asp187 and His41, thus better satisfying the tetrahedral arrangement of hydrogen bonds expected around a water molecule. Although not directly supported by the observed nuclear density for Wat409, such an interpretation would not disagree with it. Double protonation of His164 is much better supported by the nuclear density map, although one cannot exclude the possibility of the N^ɛ2^ atom being an acceptor of the deuteron from the hydroxyl group of Thr175. At this stage the exact charge state of the active site of the enzyme still requires additional data to be verified.

### 5.4. Conformation of His41 and modeling of the catalytic water molecule   

As mentioned above, the so-called catalytic water molecule plays an important role in the mechanism of catalysis by 3CL^pro^. This water molecule is located about 8 Å from Cys145 and is usually hydrogen-bonded to the peptide N atom of His41 and the N^δ1^ atoms of His41 and His164. Inspection of the hydrogen-bonding pattern of His41 and His164 is quite suggestive of the most probable rotamers of their side chains, in spite of the fact that insufficient data resolution may not permit an unambiguous decision based on the distribution of *B* factors and/or covalent geometry (Malinska *et al.*, 2015 ▸). The principle of satisfactory hydrogen-bonding patterns requires such histidine rotamers in which their N atoms are involved in optimized hydrogen bonds; however, in many PDB models of 3CL^pro^ these rotamers are inverted (despite validation alerts almost always flagging the required flips) and the catalytic water is in hydrogen-bonding contact with the C^δ2^ atoms of one or both of the histidine rings. In the re-refined structures, the histidine rotamers have been corrected to their more likely conformations.

In almost all of the 109 individual 3CL^pro^ molecules in the PDB depositions listed in Tables 1 ▸ and 2 ▸, the catalytic water molecule is modeled in unambiguous electron density. There are a few structures where there is clear electron density in the appropriate location but the water was not included in the originally deposited model. These are PDB entries 6m03, 7bro, 6xr3, molecule *A* of PDB entry 7cbt, molecule *B* of PDB entry 7jkv, molecules *A*, *C* and *D* of PDB entry 6m2n and molecules *B* and *D* of PDB entry 6xoa. The water molecules are present in molecule *B* of PDB entry 7cbt, molecule *B* of 6m2n and molecules *A* and *C* of PDB entry 6xoa. The only case without any electron density in exactly the same location is in molecule *A* of the near-atomic resolution structure with PDB code 7jkv, where the catalytic water molecule is displaced from its usual location. In both complexes of 3CL^pro^ with GRL-2420 (PDB entries 7jkv and 6xr3), this is caused by a large shift of His41 as a result of binding of this inhibitor, leading to the formation of a direct hydrogen bond between N^δ1^ of His41 and N^ɛ2^ of His164. The residual density seen in PDB entry 6xr3 and in molecule *B* of PDB entry 7jkv is most likely due to only partial (although significant) occupancy of the inhibitor.

### 5.5. Comparison of all analyzed structures of 3CL^pro^   

The availability of over 100 models of the same protein, obtained in different laboratories from different crystal forms grown in a variety of conditions, affords a unique possibility of conducting detailed structural comparisons aimed at probing the extent of structural variability enforced by crystal packing, as well as by the presence or absence of bound ligands. In order to perform such global comparisons, we have superposed the individual models of 3CL^pro^ in groups of molecules selected by various criteria (such as presence of inhibitors, space groups *etc.*) to calculate the mean positions of the C^α^ atoms representing the average (even if nonphysical) model of each group of structures. This was performed by overlapping all selected molecules onto one of them to obtain the starting mean C^α^ coordinates, and then repeating this procedure again to obtain a closer representation of the average structure. Only residues 1–300 were included in these calculations, since the orientation of the C-terminal fragment is extremely variable (see below). The selected groups are specified in Table 4 ▸, *e.g.* ‘all’, ‘free’, ‘inhibited’ *etc.*

**Figure 8:**
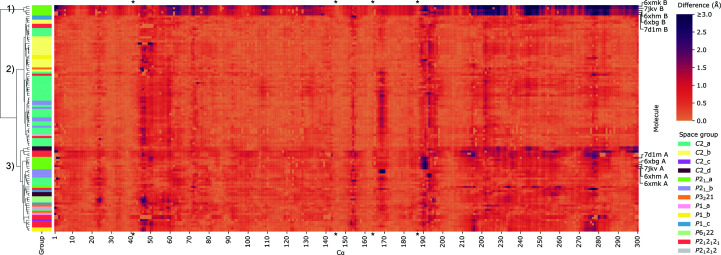
Heatmap presenting the differences (tile color) between the mean coordinates of C^α^ atoms of all of the 3CL^pro^ structures and the C^α^ coordinates for each residue (*x* axis) in each structure (*y* axis). Light tile colors indicate coordinates close to the mean, whereas dark colors represent C^α^ coordinates deviating from the mean. The dendrogram on the left presents a clustering of the structures according to their C^α^ deviations, with the colors of the leaves representing structural polymorphs (for details, see Supplementary Fig. S1). Residues directly involved in forming the catalytic site (His41, Cys145, His164 and Asp187) are marked with asterisks.

The distances of the C^α^ atoms in all 109 models from their average positions were presented in Fig. 8 ▸ as a heatmap and were used to cluster the structures using the method of Ward (1963 ▸). The clusters are presented as a dendrogram in Supplementary Fig. S1. Molecules (heatmap rows) found to be similar were placed close to each other on the *y* axis and were joined by short dendrogram branches. The lengths of these branches are proportional to the differences (distances) between clusters.

The dendrogram branch lengths delineate three distinct large clusters of similarity to the superposition mean. The first cluster, at the top of the heatmap, consists of five structures and is discussed below. The second cluster consists of structures that are very similar to the mean (light-colored heatmap tiles). These are mostly models from polymorphs *C*2_a, *C*2_b and *C*2_c with only one molecule of 3CL^pro^ in the asymmetric unit. The third cluster, in the lower half of the heatmap, consists of molecules similar to the mean up to residue 197, after which the deviations from the mean become pronounced. The clustering is also reflected in *TLSMD* analysis (Painter & Merritt, 2006 ▸), which suggested that residues 1–197, forming the larger N-terminal domain, are structurally more stable, in contrast to the smaller C-terminal domain. Importantly, all of the catalytic residues are located within the stable N-terminal domain of the enzyme.

Table 4 ▸ presents the r.m.s.d. statistics calculated for superpositions of the C^α^ atoms of a selected group of models onto the mean C^α^ positions for that group. Grouping the r.m.s.d.s of all 109 3CL^pro^ models into ‘free’ and ‘inhibited’ shows that the variation in the conformation of the protein main chain is similar in all structures. The r.m.s.d. values are all comparable, with the clear exception of the five models of chain *B* in space group *P*2_1__a.

The r.m.s.d. statistics for the relatively frequent polymorphs *C*2_a and *C*2_b show that the level of variability of protein molecules crystallized in the same space group is considerably lower than within all investigated structures. Inspection of the heatmap (Fig. 8 ▸) and Table 4 ▸ suggests that structural differences depend more on crystal packing than on the presence or absence of ligands or the temperature of the diffraction experiment.

**Figure 9:**
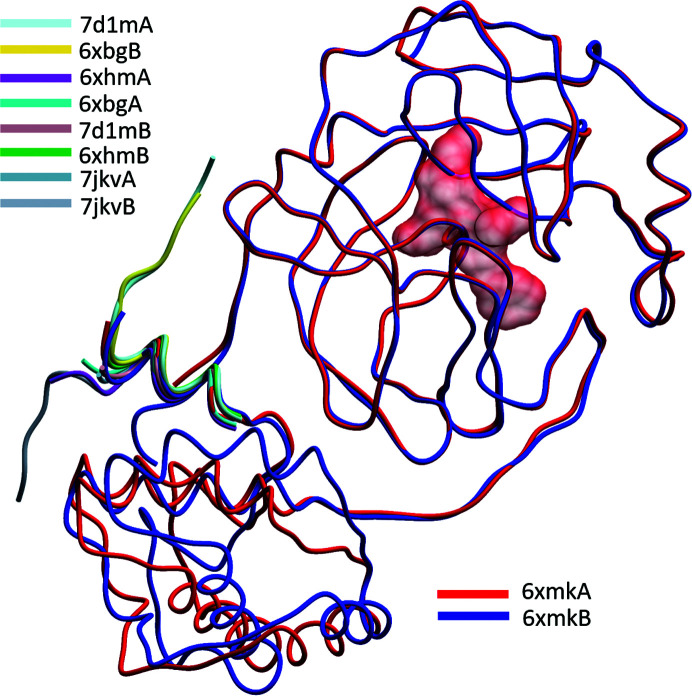
Superposition of high-resolution *P*2_1__a polymorph structure models. Superposition of the C^α^ atoms of residues 1–197 (the catalytic N-terminal domain) of 3CL^pro^. The figure illustrates the dramatic effect that intermolecular contacts can have on the plasticity of the C-terminal domain: while significant rearrangements of the C-terminal domain helices exist, as illustrated for the two most divergent models (red and blue), the helical region at the very end of the C-terminal fragment overlaps almost perfectly and maintains its orientation relative to the N-­terminal domain in all models. The final six residues diverge again and are either disordered or stabilized by intermolecular contacts. The red surface highlights the location of the binding site (ligand UAW246 in PDB entry 6xbg).

The most pronounced differences from the average ‘structure’, clearly visible in the heatmap in Fig. 8 ▸, are exhibited by five molecules labeled as the *B* chains of the structures with PDB codes 6xbg, 6xhm, 6xmk, 7d1m and 7lkv belonging to polymorph *P*2_1__a with two molecules in the asymmetric unit (Table 3 ▸). Whereas all molecules *A* in these structures are similar to the average model, molecules *B* exhibit a different domain orientation. When molecules *A* and *B* from the *P*2_1__a crystals are superposed on their N-terminal domains (residues 1–197), the polar rotation necessary to bring the smaller C-terminal domains (residues 198–306) into alignment is 18.0, 18.2, 22.4, 17.2 and 20.3°, respectively, with the hinge in the vicinity of Asp197. This significant domain rearrangement is likely to be caused by specific crystal-packing requirements. The difference in the mutual orientation of the domains is illustrated in Fig. 9 ▸.

Some details of the previously published comparisons need to be re-evaluated in view of our analysis. Lee *et al.* (2020 ▸) observed that in their structure with PDB code 7khp, in molecule *B*, representing the acyl-enzyme complex bound in the active site, the widening of the substrate-binding groove (measured as the shift of residues Gln189 and Leu167 away from each other) could be estimated as 1.5 Å relative to their structure of the free enzyme with PDB code 7jp1 or to the highest resolution structure (PDB entry 6yb7). We have compared the width of this structural region (which consists of two β-strands) in all molecules listed in Tables 1 ▸ and 2 ▸, taking into account three distances between C^α^-atom pairs facing each other on the two β-strands at the opposing sides of the groove: 164–187, 166–189 and 168–191. The results are summarized in Table 5 ▸, revealing some flexibility of the protein chains along the active-site groove. This effect is reflected in the variation (measured as r.m.s.d.) of the inter-strand distances, which increases from the pair of residues 164–187, where it is 0.24 Å, towards the pair 168–191, which is farther away from the active-site reaction center and where this value exceeds 1 Å. In addition, the stretching of the 187–191 distance is slightly more variable than that of 164–168. This result is corroborated by the scatter of the C^α^ coordinates around the average positions (Table 5 ▸). This behavior is the same in both the apo forms and the inhibitor complexes. Therefore, the particular situation noted by Lee *et al.* (2020 ▸) for just one pair of molecules cannot be given universal validity.

The increased flexibility of these two fragments of the active-site groove is also apparent in the heatmap (Fig. 8 ▸). It is likely that this phenomenon is of functional importance. The 3CL^pro^ protease functions to cleave the long polyprotein chain, translated from the viral mRNA, at 11 junctions to release the mature viral proteins. The protease cannot therefore be too specific as it has to recognize a number of substrate sequences. On the other hand, it cannot be too promiscuous as this would lead to shredding the proteins at unwanted sites.

## 6. Conclusions and outlook   

The availability of over 100 models of the same 3CL^pro^ protein, in itself an important COVID-19 drug-design target, determined by different teams within a little more than six months using several, always highly advanced, crystallo­graphic approaches, in as many as 13 polymorphic forms, provides an almost unprecedented opportunity to assess biostructural results from the point of view of quality, level of confidence and the degree of structural variability related to various experimental (for example temperature), chemical (for example crystallization conditions) or biological (for example mutations or inhibitors) factors. It is of pivotal importance that the models were not blindly accepted as raw material from the PDB, but were instead first collected in a dedicated database (https://covid-19.bioreproducibility.org), where they underwent thorough checking, validation and, if necessary, re-refinement and redeposition in the PDB.

The lessons from this large-scale meta-analysis are manifold. Firstly, they caution against the overinterpretation of small structural changes detected by biocrystallography as being of unequivocal biological significance. We have seen that such changes related to inhibitors or mutations can be masked by even larger changes caused by crystal packing. This is pointedly illustrated by a heatmap of geometrical variability of the 3CL^pro^ molecules, where the ligand-free and inhibitor complexes do not segregate but form other clusters together. Also, the expectation that room temperature will reveal features that are lost at 100 K is not supported by our results. Thus, we do not see much merit in forcing unusual experimental conditions (for example XFEL radiation) when a more mundane approach would be sufficient. In contrast, tech­niques such as neutron scattering are invaluable in providing new information.

This study would not be possible without the PDB, which has been the chief custodian of biostructural data for 50 years and one of the major driving forces that has enabled the tremendous advances in structural biology in recent decades. The PDB is continuously evolving, and in the last several years it has introduced crucial new functionalities into the validation pipeline. We find particularly important the tools focused on ligand quality and the ongoing implementation of PDB entry versioning. Nevertheless, as our experiences from this study show, there are still many possibilities for improvement that can be made to increase the quality, interpretability and reproducibility of the data gathered in the PDB. There were numerous problems with the original PDB depositions analyzed here. They include a lack of definition of ligand restraints, wrong or inconsistent ligand stereochemistry and nomenclature, poor differentiation between a polymer and its ligand, inconsistent treatment of ADPs *etc.* A separate problem, requiring an institutional solution, is the flooding of the PDB with depositions from ligand-screening campaigns such as *PanDDA*, which are not quite on a par with the rest of the database. While potentially useful for the intended purpose of fragment screening, these entries have inferior refinement status, model completeness and ligand quality metrics. Being incompatible with the conventional deposition standards, the provided maps are impossible to reproduce even with the best of intentions. In our opinion *PanDDA* data should be archived in a dedicated repository, more akin to a raw data bank, with the single data set containing the ligand deposited unaltered and clearly identified. Finally, our analysis detected, and hopefully corrected, numerous errors in the individual depositions. Apart from a lax approach to crystallographic conventions, there is the minor but annoying problem of placing the model all over the place in the unit cell (and outside). A simple run of *ACHESYM* at an early stage of structure solution should put everything on a common footing. In a number of cases there were problems with modeling of the protein portion, especially with side-chain rotamers. Even more troubling from the point of view of medical significance are modeling errors of the inhibitors, sometimes caused by wishful thinking not supported by experimental electron density. Overall, however, the problem of data misinterpretation or overinterpretation was less severe in this project than in several other structure-validation campaigns.

For biomedical researchers interested in 3CL^pro^ studies we have the following take-home messages. (i) Do not uncritically assume that the important ligand/inhibitor or other significant part of the structure deposited in the PDB is correctly modeled. Always check whether the experimental electron density is consistent with the proposed ligand conformation or look for external validation results in a dedicated database (for example, https://covid-19.bioreproducibility.org). (ii) Use graphical software for visual inspection of crucial ligand properties, such as stereochemistry or chirality. (iii) Verify ligand occupancy/ADP parameters; be alerted if the former are too low (<0.5) or the latter are too high (>80 Å^2^). (iv) Do not uncritically assume that the modeled proton distribution in the active site is correct; X-ray diffraction is very poor in this respect, and even neutron diffraction results may be questionable. (v) We also point out that even the most conserved catalytic water molecule is missing in a number of models despite the presence of unambiguous electron density: a clear warning to those who might use these models for tasks such as drug design.

Viewing the problems encountered during this work in a constructive way, we would like to make a number of recommendations for improvement of the procedures implemented by the PDB. Firstly, although the PDB site shows the overall quality percentiles of a structure, they are not available as advanced search criteria. Having these metrics directly in the search tool would enable selection of the best structure when there are many entries for the same protein. Secondly, ligand quality indicators (for example RSR and RSCC) could be introduced as sliders for major ligands (for example those over five atoms) on PDB entry pages and as advanced search criteria in order to highlight potential problems with ligand modeling prior to inspection of full validation reports. Thirdly, these and other quality metrics, such as *R* and *R*_free_, are values that should be fully reproducible. Currently, different values of these metrics are obtained from the PDB, from the primary citation and when trying to reproduce results. If a deposition was accompanied by a detailed description of the computational protocol used by the PDB when processing the data, this would greatly improve reproducibility. Fourthly, the PDB validation pipeline is currently focused on the correctness of what is in the model, but does not check for what may be missing. Adding to the validation report a gallery of screenshots of up to ten difference electron-density peaks above 5 r.m.s.d., in the same way as the electron-density fit for ligands is currently displayed, would be an excellent way of attracting the attention of both the depositors and the PDB consumers. Fifthly, as this work was focused on comparing homologous structures, we had to constantly standardize the placement of models in the unit cell and sometimes even reindex the structure factors. Although it cannot be expected that all homologous structures will always be instantly comparable, the PDB could promote (or even add to *OneDep*) a tool such as *ACHESYM* (http://achesym.ibch.poznan.pl/) that would make structural comparisons easier. Sixthly, since, as described above, the purpose and results of *PanDDA* campaigns are not directly compatible with standard PDB depositions, we suggest archiving *PanDDA* group depositions in a separate vault, where they could be compared with other fragment-screening campaigns, similarly to what is performed at https://fragalysis.diamond.ac.uk/. Finally, we would like to stress that the only way forward is to nudge the original authors to deposit prudently refined structures and to correct them when necessary. The latter aspect is greatly facilitated by the recently enabled PDB entry versioning. As the final conclusion, let us reiterate the importance of validated structural information in biomedical research (Waman *et al.*, 2020 ▸) and express hope that this analysis, conducted under the medical threat of COVID-19, will set a useful example in the case of other health emergencies.

## 7. Related literature   

The following references are cited in the supporting information for this article: Matthews (1968 ▸), Rupp (2009 ▸) and Weichenberger & Rupp (2014 ▸).

## Supplementary Material

Supplementary Tables and Figure. DOI: 10.1107/S2052252521001159/be5287sup1.pdf

Supplementary Table S1 in Excel format. DOI: 10.1107/S2052252521001159/be5287sup2.xlsx

## Figures and Tables

**Table 1:** Crystal structures of the unliganded form of 3CL^pro^

PDB code	Resolution (Å)	*R*_free_ (original/re-refined)	Space group†	Temperature	X-ray source	Remarks and references
6yb7	1.25	0.192/0.163	*C*2_a	Cryo	Synchrotron	—
6m03	2.0	0.246/0.242	*C*2_a	Cryo	Synchrotron	—
6y2e	1.75	0.222/0.221	*C*2_a	Cryo	Synchrotron	Zhang *et al.* (2020 ▸)
6xb0	1.8	0.201/0.202	*C*2_a (*I*2)	Room	Home	Kneller, Phillips, O’Neill, Tan *et al.* (2020 ▸)
6m2q	1.7	0.204/0.218	*C*2_a	Cryo	Synchrotron	Su *et al.* (2020 ▸)
6wqf	2.3	0.230/0.239	*C*2_a	Cryo	Synchrotron	Kneller, Phillips, O’Neill, Jedrzejczak *et al.* (2020 ▸)
7jp1	1.8	0.233/0.248	*C*2_a	Cryo	Synchrotron	Lee *et al.* (2020 ▸)
7bro	2.0	0.259/0.245	*C*2_a	Cryo	Synchrotron	Fu *et al.* (2020 ▸)
7cwb	1.9	0.257/0.282	*C*2_a	Room	XFEL	—
7jun	2.3/2.5	0.220	*C*2_a	Room	Home/neutron	Kneller, Phillips, Weiss *et al.* (2020 ▸)
7jvz	2.5	0.217/0.252	*C*2_a	Room	XFEL	—
7k3t	1.2	0.187/0.163	*C*2_a	Cryo	Synchrotron	—
6y84	1.39	0.200/0.186	*C*2_a	Cryo	Synchrotron	—
7jr3	1.55	0.183/0.194	*C*2_b	Cryo	Synchrotron	—
7jr4	1.55	0.180/0.186	*C*2_b	Cryo	Synchrotron	—
6xkh	1.28	0.175/0.172	*C*2_b	Cryo	Synchrotron	—
6xhu	1.8	0.246/0.247	*P*2_1__b	Room	Home	Kneller, Phillips, O’Neill, Tan *et al.* (2020 ▸)
6wtm	1.85	0.252/0.257	*P*2_1__b	Cryo	Synchrotron	Vuong *et al.* (2020 ▸)
6xkf	1.8	0.239/0.245	*P*2_1__b	Cryo	Synchrotron	—
7cwc	2.1	0.259/0.255	*P*2_1_2_1_2_1_	Room	XFEL	—
7c2y	1.91	0.262/0.233	*P*2_1_2_1_2_1_	Cryo	Synchrotron	—
7c2q	1.93	0.265/0.242	*P*2_1_2_1_2_1_	Cryo	Synchrotron	—
7jfq	1.55	0.198/0.197	*P*2_1_2_1_2_1_	Cryo	Synchrotron	—
6xoa	2.1	0.251/0.278	*P*1_c	Cryo	Synchrotron	—

† A key to the space-group symbol extended by ‘_*k*’ is given in Table 3 ▸.

**Table 2:** Crystal structures of complexes of 3CL^pro^ with ligands near the active site

PDB code	Resolution (Å)	*R*_free_ (original/re-refined)	Space group†	Temperature	X-ray source	Inhibitor‡	Remarks and references
6zrt	2.1	0.237/0.236	*C*2_a	Cryo	Synchrotron	Telaprevir	—
6xqs	1.9	0.204/0.211	*C*2_a	Room	Home	Telaprevir	Kneller, Galanie *et al.* (2020 ▸)
7k6e	1.63	0.245/0.255	*C*2_a	Cryo	Synchrotron	Telaprevir	—
7k6d	1.48	0.215/0.226	*C*2_a	Cryo	Synchrotron	Telaprevir	—
7c7p	1.74	0.216/0.193	*P*2_1_2_1_2_1_	Cryo	Synchrotron	Telaprevir	—
7c8u	2.35	0.273/0.268	*C*2_a	Cryo	Synchrotron	GC376	—
6wtj	1.9	0.235/0.242	*C*2_a	Cryo	Synchrotron	GC376/K36	Vuong *et al.* (2020 ▸)
6wtk	2.0	0.255/0.274	*C*2_a	Cryo	Synchrotron	GC373/UED	Vuong *et al.* (2020 ▸)
7c6u	2.0	0.251/0.234	*C*2_b	Cryo	Synchrotron	GC376	Fu *et al.* (2020 ▸)
6wtt	2.15	0.300/0.287	*P*3_2_21	Cryo	Synchrotron	GC376/B1S	Ma *et al.* (2020 ▸)
7d1m	1.35	0.197/0.157	*P*2_1__a	Cryo	Synchrotron	GC376	Fu *et al.* (2020 ▸), replaced 7brr
7cbt	2.35	0.292/0.272	*P*2_1__a	Cryo	Home	GC376	—
6zru	2.1	0.215/0.237	*C*2_a (*I*2)	Cryo	Synchrotron	Boceprevir	—
7brp	1.8	0.240/0.219	*P*2_1__b	Cryo	Synchrotron	Boceprevir	Fu *et al.* (2020 ▸)
7c6s	1.6	0.222/0.166	*C*2_b	Cryo	Synchrotron	Boceprevir	Fu *et al.* (2020 ▸)
6wnp	1.44	0.196/0.158	*C*2_b	Cryo	Synchrotron	Boceprevir	—
7k40	1.35	0.192/0.183	*C*2_a	Cryo	Synchrotron	Boceprevir	—
7com	2.25	0.246/0.249	*P*2_1_2_1_2_1_	Cryo	Synchrotron	Boceprevir	—
6xqu	2.2	0.234/0.248	*C*2_a (*I*2)	Room	Home	Boceprevir	Kneller, Galanie *et al.* (2020 ▸)
6xqt	2.3	0.277/0.249	*P*2_1__b	Room	Home	Narlaprevir	Kneller, Galanie *et al.* (2020 ▸)
7d1o	1.78	0.249/0.240	*C*2_a	Cryo	Synchrotron	Narlaprevir	—
7jyc	1.79	0.213/0.214	*C*2_a	Cryo	Synchrotron	Narlaprevir	—
6xch	2.2	0.237/0.240	*C*2_a (*I*2)	Room	Home	Leupeptin	Kneller, Galanie *et al.* (2020 ▸)
6yz6	1.7	0.216/0.238	*C*2_a	Cryo	Synchrotron	Leupeptin	—
6xfn	1.7	0.228/0.229	*C*2_a	Cryo	Synchrotron	UAW243	Sacco *et al.* (2020 ▸)
6xbi	1.7	0.217/0.226	*P*1_a	Cryo	Synchrotron	UAW248	Sacco *et al.* (2020 ▸)
6xbh	1.6	0.221/0.207	*C*2_a	Cryo	Synchrotron	UAW247	Sacco *et al.* (2020 ▸)
6xbg	1.45	0.206/0.181	*P*2_1__a	Cryo	Synchrotron	UAW246	Sacco *et al.* (2020 ▸)
6xa4	1.65	0.239/0.248	*C*2_a	Cryo	Synchrotron	UAW241	Sacco *et al.* (2020 ▸)
7c8b	2.2	0.230/0.230	*C*2_b	Cryo	Synchrotron	Z-VAD-FMK	—
7bqy	1.7	0.226/0.238	*C*2_b	Cryo	Synchrotron	N3	Jin, Du *et al.* (2020 ▸)
6lu7	2.16	0.235/0.225	*C*2_b	Cryo	Synchrotron	N3	Jin, Du *et al.* (2020 ▸)
7buy	1.6	0.201/0.205	*C*2_b	Cryo	Synchrotron	Carmofur	Jin, Zhao *et al.* (2020 ▸)
7ju7	1.6	0.192/0.171	*C*2_b	Cryo	Synchrotron	Mastinib	—
7jkv	1.25	0.177/–	*P*2_1__a	Cryo	Synchrotron	GRL2420	Hattori *et al.* (2021 ▸)
6xr3	1.45	0.187/0.184	*C*2_b	Cryo	Synchrotron	GRL2420	—
6w63	2.1	0.221/0.250	*P*2_1_2_1_2	Cryo	Synchrotron	X77	—
6w79	1.46	0.177/0.154	*C*2_c	Cryo	Synchrotron	X77	—
6yt8	2.05	0.233/0.258	*C*2_a	Cryo	Synchrotron	PK8	Günther *et al.* (2020 ▸)
6z2e	1.7	0.243/0.255	*P*6_1_22	Cryo	Synchrotron	Q5T	—
7d3i	2.0	0.209/0.207	*C*2_a	Cryo	Synchrotron	MI-23	—
6lze	1.5	0.199/0.187	*C*2_b	Cryo	Synchrotron	11A	Dai *et al.* (2020 ▸)
6m0k	1.5	0.193/0.178	*C*2_b	Cryo	Synchrotron	11B	Dai *et al.* (2020 ▸)
6m2n	2.2	0.254/0.271	*P*1_b	Cryo	Synchrotron	3WL	Su *et al.* (2020 ▸)
6xb2	2.1	0.257/0.282	*C*2_a (*I*2)	Room	Home	NEN	Kneller, Phillips, O’Neill, Tan *et al.* (2020 ▸)
6xb1	1.8	0.202/0.210	*C*2_a (*I*2)	Room	Home	NEN	Kneller, Phillips, O’Neill, Tan *et al.* (2020 ▸)
6xhm	1.41	0.210/0.192	*P*2_1__a	Cryo	Synchrotron	V2M	Hoffman *et al.* (2020 ▸)
6xmk	1.7	0.212/0.214	*P*2_1__a	Cryo	Synchrotron	7J	Rathnayake *et al.* (2020 ▸)
6y2f	1.95	0.219/0.206	*C*2_b	Cryo	Synchrotron	O6K	Zhang *et al.* (2020 ▸)
6y2g	2.2	0.247/0.240	*P*2_1_2_1_2_1_	Cryo	Synchrotron	O6K	Zhang *et al.* (2020 ▸)
6ynq	1.8	0.226/0.247	*C*2_a	Cryo	Synchrotron	P6N	Günther *et al.* (2020 ▸)
6yvf	1.6	0.208/0.243	*C*2_a	Cryo	Synchrotron	AZD6482	Günther *et al.* (2020 ▸)
7c8r	2.3	0.261/0.261	*P*6_1_22	Cryo	Synchrotron	TG0203770	—
7c8t	2.05	0.243/0.252	*P*6_1_22	Cryo	Synchrotron	TG0205221	—
7cx9	1.73	0.209/0.188	*C*2_b	Cryo	Synchrotron	INZ-1	—
7joy §	2.0	0.252/–	*C*2_d	Cryo	Synchrotron	Self	Lee *et al.* (2020 ▸)
7khp §	1.95	0.248/–	*C*2_d	Cryo	Synchrotron	Self	Lee *et al.* (2020 ▸), replaced 7jox

† A key to the space-group symbol extended by ‘_*k*’ is given in Table 3 ▸.

‡ Inhibitors are identified by their common names or, if such a name was not listed in the PDB deposition, by the CCP4 code used by the PDB.

§ The original PDB depositions 7jox and 7joy were standardized, re-refined and redeposited or updated in the PDB by the original authors (Lee *et al.*, 2020 ▸).

**Table 3:** Key to different crystal forms of 3CL^pro^ with approximate unit-cell parameters and solvent content

Space group†	*a* (Å)	*b* (Å)	*c* (Å)	β or α/β/γ for *P*1 (°)	Molecules in asymmetric unit	Solvent content (%)
*C*2_a	114	53	45	101	1	39
*C*2_b	98	81	52	115	1	55
*C*2_c	109	81	53	104	1	45
*C*2_d	124	80	63	90	2	47
*P*2_1__a	55	99	59	108	2	45
*P*2_1__b	46	54	114	101	2	54
*P*2_1_2_1_2_1_	68	100	104		2	55
*P*2_1_2_1_2	45	64	107		1	46
*P*6_1_22	104	104	90		1	42
*P*3_2_21	101	101	160		3	47
*P*1_a	47	55	62	62/61/80	2	38
*P*1_b	64	68	95	74/78/66	4	54
*P*1_c	63	68	78	78/90/73	4	46

† In most cases the space-group symbol is extended by ‘_*k*’, where ‘*k*’ designates a distinct polymorph within the same space group.

**Table:** (*a*) Statistics of the average r.m.s.d. of C^α^ atoms in selected groups of molecules from their mean position in each residue.

No.	Molecules	No. of models	Average r.m.s.d.	Minimum r.m.s.d.	Maximum r.m.s.d.	
1	All	109	0.53	0.28	1.86	6xmk chain *B*
2	Free	33	0.46	0.27	1.03	7cwc chain *A*
3	Inhibited	76	0.55	0.31	1.84	6xmk chain *B*
4	Inhibited excluding *P*2_1__a	66	0.47	0.21	0.86	7khp chain *B*
5	Free *C*2_a	13	0.28	0.18	0.54	7bro chain *A*
6	Free *C*2_b	3	0.27	0.21	0.30	7jr4 chain *A*
7	Inhibited C2_a	20	0.36	0.26	0.45	7k6e chain *A*
8	Inhibited *C*2_b	10	0.29	0.21	0.44	7c8u chain *A*

**Table:** (*b*) R.m.s.d. values resulting from comparing the average C^α^ positions in separate groups of molecules.

No.		1	2	3	4	5	6	7
1	All	—						
2	Free	0.18	—					
3	Inhibited	0.08	0.25	—				
4	Inhibited excluding *P*2_1__a	0.21	0.30	0.22	—			
5	Free *C*2_a	0.32	0.22	0.37	0.43	—		
6	Free *C*2_ b	0.38	0.44	0.40	0.27	0.60	—	
7	Inhibited *C*2_a	0.22	0.24	0.23	0.30	0.24	0.49	—
8	Inhibited *C*2_b	0.21	0.30	0.22	0.01	0.43	0.27	0.30

**Table 5:** Variations in the width of the active-site groove in the analyzed molecules

	C^α^–C^α^ distance (Å)			Distance from the average C^α^ position (Å)					
Residues	164–187	166–189	168–191	164	166	168	187	189	191
Free (33 molecules)									
Average	7.18	9.69	7.65	0.93	1.09	1.40	1.13	1.37	1.49
R.m.s.d. (average)	0.20	0.74	0.87	0.05	0.14	0.17	0.13	0.43	0.37
Minimum	6.94	8.96	6.57	0.79	0.71	1.03	0.85	0.65	0.81
Maximum	7.80	11.36	9.22	1.02	1.40	1.82	1.46	2.34	2.23
Inhibited (76 molecules)									
Average	7.28	10.10	8.23	0.48	0.63	0.98	0.60	0.97	1.45
R.m.s.d. (average)	0.24	0.53	1.25	0.10	0.16	0.36	0.17	0.38	0.57
Minimum	6.77	9.10	6.11	0.26	0.30	0.51	0.23	0.14	0.52
Maximum	7.88	11.44	11.11	0.79	1.03	2.26	1.01	1.97	2.90
