# Supplementary material for: Crystallographic models of SARS-CoV-2 3CLpro: in-depth assessment of structure quality and validation
Source: IUCrJ. 2021 Feb 9;8(Pt 2):238–56. doi: 10.1107/S2052252521001159 (PMC7924243; doi:10.1107/S2052252521001159)
Supplement: Supplementary file 1 [file m-08-00238-sup1.pdf]

IUCrJ

Volume 8 (2021)

Supporting information for article:

Crystallographic models of SARS-CoV-2 3CLpro protease: in-depth assessment of structure quality and validation

Mariusz Jaskolski, Zbigniew Dauter, Ivan Shabalin, Miroslaw Gilski, Dariusz Brzezinski, Marcin Kowiel, Bernhard Rupp and Alexander Wlodawer

Supplementary Table S1. External Table in sortable MS Excel Table format containing additional data items used in or useful for further analysis. Using Custom Sort from the sort and filter menu allows for example to sort by space group and then by resolution. The Table can also be exported in other machine-readable formats. The non-tabular items are explanatory or ancillary only, the charts show examples for some statistical analysis.

PDB ID	4-character PDB identifier
Resol (Å)	reported resolution in PDB file
a, b, c (Å), alpha, beta, gamma (deg)	unit cell constants from CRYST1 record
Space group	space group symbol from CRYST1 plus polymorph indicator [*]
Vol (Å^3)	unit cell volume, computed as in (Rupp, 2009)
N (asu)	number of molecules in asymmetric unit, from PDB file
Z	multiplicity of general position for given space group
Vm (Å^3/Da)	Matthews coefficient (Matthews, 1968) calculated as in (Weichenberger & Rupp, 2014)
Vs (%)	solvent content in % , calculated as in (Weichenberger & Rupp, 2014)
Rfree original	reported R_{free} from PDB file
Rfree re-refined	R_{free} afer re-refinement per protocol listed in methods section
Temp	data collection temperature, cryogenic or room temperature
Source	type of X-ray or neutron source; sync: Synchrotron, home: Laboratory X-ray source, N: Neutron spallation source, XFEL: X-ray free electron laser
Inhibitor	trade name of inhibitor; self: self-inhibition by protease C terminus; 0: unliganded
Link	type of linkage; linked protease residue Cys145
LIG	3-letter HET identifier from PDB (multiple for inhibitory peptide analogs)
Tag/Mut	tags and mutants indicated *via* sequence number and single letter identifier extracted from SEQADV
pH	crystallization pH parsed from REMARK 280 if available [**]
Precip	presumed primary precipitant, parsed from REMARK 280, encoded as %Molecular_weight of PEG [**]
DMSO	percentage of DMSO added, if reported [**]
Buffer	main buffer component extracted from REMARK 280; encoded as molarity and buffer type abbreviation (self-explanatory) [**]
Remarks and reference	self-explanatory

[*] Polymorph indicator _a, _b, _c indicates a non-isomorphous polymorph of the same space group. (*I*2) indicates that, for consistency, the original *I*2 unit cell was transformed to polymorph *C*2_a.

[**] Crystallization condition reporting is not standardized in any machine-readable form; this table includes only a limited subjective subset.

Supplementary Table S2. Selected geometrical parameters of 3CLpro models as originally deposited in the PDB. The values in each row are: torsion angle N-Cα-Cβ-Sγ of Cys145 [(χ1(C145)], torsion angles Cα-Cβ-Cγ-Nδ1 of His41 [(χ2 (H41)] and His164 [(χ2 (H164)], distance between the Nδ1 atoms of His41 and His164 [δ(Nδ1)], distances from the "catalytic water" to atoms N of Cys145, Nδ1 of His41, Nδ1 of His164, and Oδ1/2 of Asp187. For covalent complexes by with oligopeptidic inhibitors, H-bond lengths between the hemiacetal/hemiketal oxygen atom and the N atom of Cys145 or Nε2 atom of His41 are given. "dens" means that there is no catalytic water in the PDB model, but there is clear electron density in an appropriate place. Structures with unusual features, discussed in the main text, are marked with an asterisk.

PDB mol.	χ1(C145)	χ2(H41)	χ2(H164)	d(Nδ1)	distance from catalytic water to	N145	Nδ41	Nδ164	OD187	distance from aldo/keto O to N145 or Nε41
6yb7 A	-63.2	68.38 *	-120.35	4.18	HOH644	3.05	3.10	2.87	2.75	
6m03 A	-56.2	71.61 *	63.34 *	4.07	dens	3.06	2.77	2.83	3.10	
6y2e A	-68.4	-119.20	-122.43	4.34	HOH582	3.07	2.98	2.94	2.98	
6xb0 A	-59.2 *	-114.40	-117.24	4.36	HOH571	3.20	2.93	2.92	2.88	
6m2q A	-63.8	-104.92	-114.70	4.30	HOH518	3.07	2.90	2.92	2.94	
6wqf A	-62.9	-104.62	-105.45	4.22	HOH417	3.14	2.79	3.02	2.94	
7jp1 A	-60.1	-137.12	-118.27	4.27	HOH477	3.00	3.13	2.99	2.78	
7bro A	-93.2	77.59 *	-109.97	4.43	dens	3.18	2.81	3.06	3.12	
7cwb A	-58.5	-101.98	-116.20	4.36	HOH441	3.19	2.85	2.88	2.81	
7jun A	-48.2	-92.18	-116.79	4.47	DOD409	3.15	2.64	3.10	2.78	
7jvz A	-60.9	-107.70	-110.71	4.37	HOH429	3.29	3.19	2.94	3.03	
7k3t A	-62.4	-120.20	-123.02	4.31	HOH601	3.09	3.07	2.86	2.75	
6y84 A	-63.0	71.49 *	-118.26	4.14	HOH604	3.07	3.12	2.85	2.72	
7jr3 A	-68.4	60.21 *	-114.12	4.19	HOH492	3.00	3.36	2.96	2.77	
7jr4 A	-65.9	66.03 *	-115.70	4.13	HOH628	3.09	3.17	2.94	2.87	
6xkh A	-47.3 *	-126.82	-123.18	4.06	HOH602	2.95	3.17	3.00	2.84	
6xhu A	-59.5	-103.08	-119.76	4.40	HOH429A	3.15	2.93	2.88	2.76	
6xhu C	-60.5	-103.08	41,67 *	4.36	HOH441C	3.27	2.87	2.85	2.80	
6wtm A	-63.6	78.63 *	-115.41	4.28	HOH460A	3.15	3.09	2.95	2.79	
6wtm B	-62.2	-115.41	-115.64	4.27	HOH455B	3.16	2.97	2.83	2.97	
6xkf A	-59.6	-104.73	-117.20	4.33	HOH557A	3.06	2.94	2.93	2.97	
6xkf B	-65.0	-114.75	-114.81	4.30	HOH536B	3.17	2.92	2.90	2.97	
7cwc A	-60.8	74.17 *	-112.43	4.24	HOH423A	3.29	3.30	2.88	3.10	
7cwc B	-69.3	69.86 *	-110.10	4.36	HOH421B	3.29	3.06	3.01	3.17	
7c2y A	-72.2	-116.34	-121.68	4.31	HOH346A	3.03	3.04	2.93	2.76	
7c2y B	-74.4	67.76 *	-116.23	4.24	HOH347B	2.89	3.04	3.04	2.96	
7c2q A	-69.2	68.82 *	-128.52	4.35	HOH334A	3.02	3/05	2.92	2.76	
7c2q B	-79.9	63.79 *	-118.60	4.33	HOH337B	3.00	3.16	2.95	2.89	
7jfq A	-62.4	-105.10	-117.65	4.25	HOH513B	3.34	2.62	3.01	2.67	
6xoa A	-79.7 *	80.77 *	67.60 *	4.14	HOH512A	2.93	3.09	3.16	3.17	
6xoa B	-66.4 *	79.51 *	69.73 *	3.92	dens	3.19	3.00	3.04	2.98	
6xoa C	-77.3 *	69.73 *	65.45 *	4.28	HOH515C	3.18	3.25	3.07	3.14	

6xoa	D	-54.8	*	65.45	*	73.55	*	3.66		dens	2.93	2.57	3.03	3.18					
6zrt	A	-70.1		-105.80		-119.57		4.14		HOH708	3.41	2.55	2.88	2.81	S	keto	Nε2 41	2.59	
6xqs	A	-70.0		-96.31		-120.48		4.02		HOH524	3.36	2.64	2.94	2.87	S	keto	Nε2 41	2.53	
7k6e	A	-63.4		-102.41		-123.45		4.13		HOH520	3.33	2.73	2.90	2.83	S	keto	Nε2 41	2.47	
7k6d	A	-64.7		-97.71		-121.36		4.15		HOH519	3.36	2.72	2.96	2.77	S	keto	Nε2 41	2.49	
7c8u	A	-77.5		-113.38		-112.68		4.22		HOH520	3.25	3.06	2.88	2.76	S	aldo	N 145	3.28	
6wtj	A	-78.9		-95.88		-114.50		4.29		HOH528	3.12	2.88	2.93	2.92	S	aldo	N 145	3.04	
6wtk	A	-78.6		-103.40		-113.87		4.12		HOH512	3.17	2.75	2.80	2.73	S	aldo	N 145	3.10	
6zru	A	-73.4		-111.87		-117.15		4.04		HOH 10	3.32	2.73	2.94	2.85	S	keto	Nε2 41	2.57	
7k40	A	-71.8		-99.85		-122.59		4.18		HOH581	3.42	2.72	2.86	2.77	S	keto	Nε2 41	2.51	
6xqu	A	-78.5		-98.28		-114.60		4.18		HOH516	3.45	2.68	2.91	2.87	S	keto	Nε2 41	2.42	
7d1o	A	-63.1		-107.29		-116.77		4.10		HOH563	3.48	2.87	2.88	3.04	S	keto	Nε2 41	2.55	
7jyc	A	-70.1		-102.60		-121.18		4.02		HOH572	3.42	2.71	2.87	2.73	S	keto	Nε2 41	2.45	
6xch	A	-73.9		-103.68		-114.32		4.31		HOH426	3.31	2.68	3.02	3.09	S	aldo	N 145	3.09	
6yz6	A	-64.6		-104.09		-116.57		4.34		HOH667	2.45	2.85	2.98	2.88	S/R	aldo	N145/41	2.83/2.56	
6xfn	A	34.4	*	-95.60		-122.83		4.11		HOH640	3.27	2.68	2.98	2.76	R	keto	Nε2 41	2.41	*
6xbh	A	-71.3		59.89	*	-121.31		4.13		HOH698	3.04	3.31	2.96	2.82	S	aldo	N 145	2.83	
6xa4	A	-73.3		67.71	*	-122.99		4.21		HOH698	3.04	3.03	2.90	2.92	S	aldo	N 145	2.89	
6yt8	A	-60.4		-107.60		-113.26		4.30		HOH564	3.09	2.70	2.93	3.26		non-peptidic			
7d3i	A	-68.4		-97.62		-121.36		4.16		HOH603	3.19	2.75	2.88	2.75	S	aldo	N 145	2.89	
6xb2	A	-67.7		-124.63		-115.67		4.52		HOH532	3.13	3.09	2.90	2.91		non-peptidic			
6xb1	A	-63.2	*	-120.03		-118.34		4.32		HOH570	3.20	3.02	2.87	2.84		non-peptidic			
6ynq	A	-76.3		53.96		-108.66		4.31		HOH666	3.02	3.20	2.95	3.13		non-peptidic			
6yvf	A	-66.4		-121.02		-114.88		4.20		HOH647	3.07	3.07	2.94	2.82		non-covalent			
7c6u	A	-69.6		-96.69		-103.07		4.15		HOH583	3.21	2.84	2.93	3.12	S	aldo	N 145	2.72	
7c6s	A	-67.5		-103.29		-117.52		4.12		HOH609	3.31	2.76	2.89	2.86	S	keto	Nε2 41	2.54	
6wnp	A	-67.7		-107.17		-124.73		4.17		HOH604	3.18	2.79	2.97	2.85	S	keto	Nε2 41	2.60	
7c8b	A	-70.2		-125.41		-117.60		4.20		HOH589	3.24	2.89	2.95	2.77		non-peptidic			
7bqy	A	-71.0		-110.78		-115.12		4.14		HOH570	3.27	2.84	2.83	2.80		non-peptidic			
6lu7	A	-81.0		-106.83		-109.11		4.13		HOH445	3.32	2.80	3.08	2.90		covalent adduct			
7buy	A	-63.8	*	66.95	*	-119.63		4.10		HOH571	3.00	3.25	2.92	2.77		thioester			
7ju7	A	-67.2		-117.81		-118.17		4.27		HOH595	3.02	3.05	2.95	2.83		non-covalent			
6xr3	A	-70.8		93.57	*	-120.92		2.91	*	dens	2.65	1.93	3.04	2.69	S	aldo	N 145	2.91	
6lze	A	-72.3		64.56	*	-119.88		4.09		HOH584	2.97	3.26	2.93	2.79	S	aldo	N 145	2.88	
6m0k	A	-69.5		70.70	*	-115.22		4.09		HOH592	2.93	3.08	2.99	2.87	S	aldo	N 145	2.83	
6y2f	A	-72.6		-113.11		-116.68		3.94		HOH654	3.27	2.76	2.86	2.83	S	keto	Nε2 41	2.68	
7cx9	A	-154.8	*	-110.33		-117.56		7.12	*	HOH619	3.03	5.13	2.94	2.78		thioester			
6w79	A	-53.7		68.94	*	-120.78		4.08		HOH623	3.03	3.10	2.97	2.75		non-covalent			
7joy	A	ALA		-100.30		-113.48		4.19		HOH417A	3.27	2.79	3.03	2.64		no Cys			
7joy	B	ALA		-100.32		-113.42		4.16		HOH421B	3.32	2.86	2.90	2.65		no Cys			
7khp	A	-62.1		-114.30		-116.37		4.22		HOH503A	3.26	3.29	3.07	2.43		no complex			
7khp	B	-65.2	*	-116.37		-113.04		4.18		HOH537B	2.99	2.83	3.08	2.88		thioester			
7d1m	A	-73.9		-103.02		-115.72		4.17		HOH626A	3.22	2.85	3.09	2.80	S/R	aldo	N145/41	2.88/2.85	

7d1m	B	-72.3	-115.72	-117.91	4.24	HOH631B	3.21	2.90	2.87	2.78	S/R	aldo	N145/41		2.87/2.83
7cbt	A	-73.2	62.16 *	-116.04	4.16	dens	3.19	2.85	2.90	3.02	S	aldo	N	145	3.16
7cbt	B	-72.9	72.38 *	-121.52	4.32	HOH549B	3.03	3.05	3.03	2.92	S	aldo	N	145	3.04
6xbg	A	-76.4	-91.30	-118.23	3.96	HOH450A	3.33	2.73	2.95	2.78	S	keto	Nε2	41	2.59
6xbg	B	-75.9	-118.23	-118.75	4.03	HOH663B	3.34	2.84	2.89	2.70	S	keto	Nε2	41	2.64
7jkv	A	-75.4	90.41 *	-115.90	2.82 *	---------					S	aldo	N	145	2.89
7jkv	B	-73.4	88.92 *	-115.67	2.83 *	dens	3.30	1.92	3.25	2.87	S	aldo	N	145	2.94
6xhm	A	-73.9	87.09 *	-120.15	3.98	HOH9150A	3.33	2.81	2.91	2.74	R	keto	Nε2	41	2.95
6xhm	B	-73.8	-120.15	-121.50	4.09	HOH9133B	3.34	2.80	2.90	2.86	R	keto	Nε2	41	2.95
6xmk	A	-73.4	-114.16	-114.86	4.19	HOH586A	3.06	2.95	2.99	2.98	S	aldo	N	145	2.95
6xmk	B	-74.5	-106.78	-120.98	4.24	HOH591B	3.14	2.94	2.92	2.81	S	aldo	N	145	2.87
7brp	A	-70.5	-102.00	-112.55	4.05	HOH559A	3.41	2.72	2.96	2.99	S	keto	Nε2	41	2.65
7brp	B	-68.3	-112.55	-112.99	4.12	HOH575B	3.50	2.75	2.96	2.92	S	keto	Nε2	41	2.58
6xqt	A	-70.4	-103.72	70.32 *	4.05	HOH503A	3.49	2.45	3.09	3.08	S	keto	Nε2	41	2.78
6xqt	B	-73.2	-93.87	72.64 *	3.98	HOH504B	3.41	2.47	3.13	2.89	S	keto	Nε2	41	2.66
6xbi	A	-75.1	-91.17	-119.24	3.91	HOH625A	3.16	2.61	2.93	2.88	S	keto	Nε2	41	2.69
6xbi	B	-73.2	-99.08	-116.19	3.96	HOH651B	3.37	2.71	2.81	2.77	S	keto	Nε2	41	2.74
6m2n	A	-83.0	67.59 *	-108.90	4.29	dens	2.95	2.98	3.06	3.35		non-covalent			
6m2n	B	-89.7	60.28 *	-109.76	4.35	HOH501B	3.05	3.11	3.13	2.31		non-covalent			
6m2n	C	-83.5	60.35 *	50.25 *	4.29	dens	2.98	2.84	3.30	2.99		non-covalent			
6m2n	D	-81.7	54.11 *	-118.61	4.23	dens	2.90	3.05	3.13	2.99		non-covalent			
7c7p	A	-64.5	-105.55	-128.89	4.09	HOH535A	3.23	2.90	2.96	2.62	S	keto	Nε2	41	2.44
7c7p	B	-63.7	-128.89	-115.64	4.01	HOH567B	3.23	3.05	2.95	2.74	S	keto	Nε2	41	2.55
7com	A	-70.8	-103.07	-118.26	4.15	HOH566A	3.24	2.76	2.95	2.79	S	keto	Nε2	41	2.46
7com	B	-63.8	-118.26	-116.03	4.15	HOH566A	3.21	2.76	2.95	2.79	S	keto	Nε2	41	2.44
6y2g	A	-73.2	-106.66	-118.95	4.03	HOH535B	3.24	2.78	2.98	2.72	S	keto	Nε2	41	2.81
6y2g	B	-79.1	-100.84	-113.78	3.79	HOH560A	3.22	2.82	2.94	2.94	S	keto	Nε2	41	2.92
6w63	A	-48.6	62.85 *	-116.18	4.12	HOH576	3.10	3.33	2.88	2.73		non-covalent			
6wtt	A	-69.5	-104.24	-105.93	4.08	HOH557A	3.16	2.94	2.82	3.03	S	aldo	N	145	2.93
6wtt	B	-65.2	83.07 *	57.48 *	4.67	HOH519B	3.11	3.60	3.12	2.61	S	aldo	N	145	2.82
6wtt	C	-74.2	-111.99	-117.44	4.42	HOH517C	3.37	2.91	3.19	2.69	R	aldo	N	145	2.91 *
6z2e	A	-71.9	-106.60 *	-118.99	4.25	HOH571	3.42	2.72	2.92	2.73		covalent, not peptidic			
7c8r	A	-71.9	55.55 *	73.78 *	7.25 *	HOH546	3.14	5.53	3.13	2.89		covalent, not peptidic			
7c8t	A	-70.0 *	59.35 *	-128.06	4.46	HOH572	3.01	3.39	3.08	2.87		thioester			

Supplementary Figure S1. Detailed view of the clustering presented in Figure 8 of the main text. The dendrogram presents the hierarchical clustering of all 3CLpro structures using Ward's method (Ward, 1963). Colors at the leaves represent structural polymorphs. The lengths of the dendrogram branches are proportional to the difference between clusters.

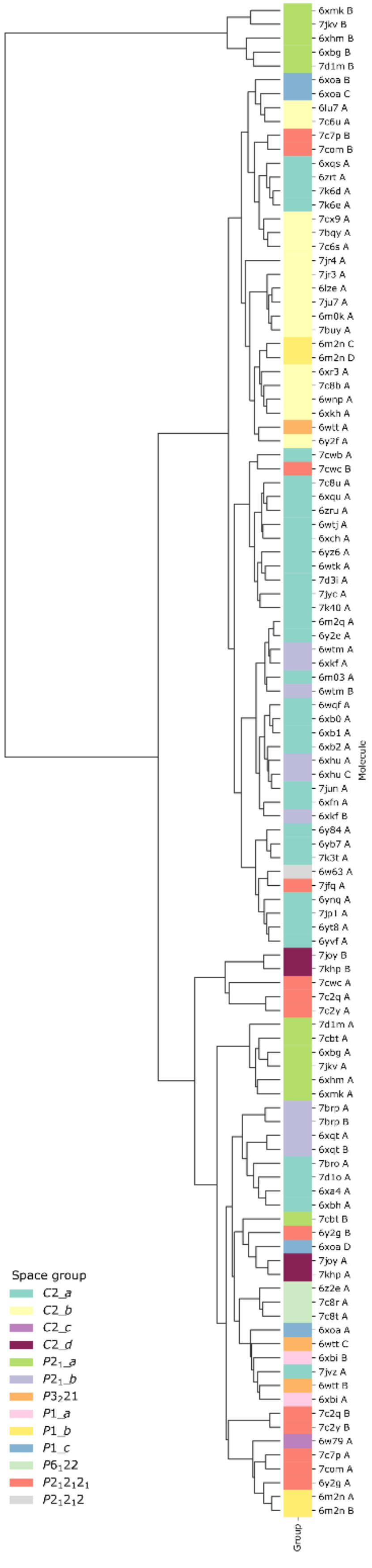

References

Matthews, B. W. (1968). *J. Mol. Biol.* **33**, 491-497.

Rupp, B. (2009). *Biomolecular Crystallography Principles, Practice, and Applicaton to Structural Biology*, 1st ed. New York: Garland Science Titles.

Ward, J. H. (1963). *J. Amer. Stat. Assoc.* **58**, 236-244.

Weichenberger, C. X. & Rupp, B. (2014). *Acta Cryst.* D**70**, 1579-1588.
